# Unlock the Potential of *Pichia* in Coffee: Knowledge Transfer From Fermented Beverages

**DOI:** 10.1111/1541-4337.70607

**Published:** 2026-08-23

**Authors:** Hosam Elhalis

**Affiliations:** ^1^ Global Technology Centre, AB Mauri Sydney Australia; ^2^ Food Science and Technology, School of Chemical Engineering The University of New South Wales Sydney Australia

## Abstract

Considerable research has focused on non‐*Saccharomyces* yeasts to explore their biochemical potential and genetic diversity beyond the capabilities of the conventional starter culture, *Saccharomyces cerevisiae*. Among these, species of the genus *Pichia* have emerged as promising candidates in diverse food fermentation systems. Although studies on the application of *Pichia* species in coffee fermentation remain limited, existing evidence indicates positive outcomes, including improved regulation of fermentation kinetics and enhanced coffee bean quality. This review critically examines the occurrence of *Pichia* species and synthesizes current knowledge of their functional traits, drawing on findings from a wide range of fermented beverages, including wine and beer. Several yeast species previously classified within the genus *Pichia*, including *Pichia kudriavzevii*, *Pichia kluyveri*, *Pichia fermentans*, *Wickerhamomyces anomalus* (formerly *Pichia anomala*), *Meyerozyma guilliermondii* (formerly *Pichia guilliermondii*), and *Debaryomyces hansenii* (formerly *Pichia hansenii*), exhibit robust fermentation performance across diverse processing conditions, including submerged systems. These yeasts contribute to microbial ecosystem stability, substrate transformation, and the enhancement of sensory complexity in fermented products. At the species level, distinct functional specializations, such as elevated ester production, pectinolytic activity, and tolerance to extreme physicochemical stress, enable targeted modulation of aroma and flavor profiles. Overall, the evidence presented in this review highlights *Pichia* species as underexploited yet valuable microbial resources capable of improving fermentation control, expanding biochemical and sensory diversity, and enhancing product quality in washed coffee processing.

## 1 Introduction

Coffee ranks among the most widely consumed beverages worldwide, constituting the second most traded commodity on international markets. Post‐harvest processing of coffee entails the conversion of harvested cherries into green beans through removal of the fruit's outer layers (Elhalis, Cox, et al. 2023a; Soares et al. 2025; Vinícius de Melo Pereira et al. 2017). Coffee processing broadly follows two principal pathways: the wet (washed) process and the natural (dry) process. In the wet process, the pulp is mechanically removed, and the beans undergo fermentation, followed by washing and drying; the natural dry process, whereby intact cherries are dried before hulling, permits flavor development within the fruit.

Within washed processing, fermentation constitutes a critical step in mucilage degradation (Janne Carvalho Ferreira et al. 2023; Revelo‐Romo et al. 2025) and is a key determinant of the sensory quality and economic value of the final beverage (Elhalis, Cox, et al. 2023a; Elhalis et al. 2021c; Haile and Kang 2019c; Janne Carvalho Ferreira et al. 2023; Shen et al. 2025).

Non‐*Saccharomyces* yeasts are consistently isolated from coffee fermentations and, in several systems, attain high relative abundance. Among these, *Pichia* species are recurrently detected across diverse processing conditions (Elhalis, Cox, et al. 2023b; Elhalis, Cox, Frank, et al. 2020; Elhalis et al. 2021a; Haile and Kang 2019a; Martins et al. 2019; Massawe and Lifa 2010; Prakash et al. 2022). The genus is characterized by tolerance to environmental stressors, metabolic versatility, capacity for aromatic compound production, biocontrol activity, and potential probiotic properties (Vicente et al. 2021; Walker 2011). In wine and beer fermentation, *Pichia* species enhance aromatic complexity, produce substrate‐modifying enzymes, and suppress spoilage organisms. Nevertheless, commercial application remains largely confined to *Pichia kluyveri*, despite the breadth of beneficial traits reported for the genus (Choi et al. 2017; Ganapathiwar and Bhukya 2023; Passoth et al. 2006; Petersson and Schnürer 1995; Vicente et al. 2021; Walker 2011). Despite their consistent presence, the functional roles of *Pichia* in coffee fermentation remain underexplored. Enzyme–metabolite–sensory relationships specific to coffee are largely unverified, and applied questions surrounding inoculation strategy, coculture design, and strain‐level characterization remain unresolved. These gaps impede the rational deployment of *Pichia*‐based starter cultures in commercial coffee processing.

While prior reviews have broadly addressed coffee microbial ecology (Elhalis, Cox, et al. 2023a; Haile and Kang 2019b; Huch and Franz 2015; Janne Carvalho Ferreira et al. 2023; Pereira et al. 2022; Shen et al. 2025; Soares et al. 2025; Vinícius de Melo Pereira et al. 2017) and non‐*Saccharomyces* yeasts in beverage fermentation (Basso et al. 2016; Batista et al. 2026; Johnson 2013; Jolly et al. 2017; Vicente et al. 2021; Walker 2011), none has systematically examined *Pichia* as a focal group within coffee processing or consolidated cross‐system functional knowledge for coffee‐specific translation. This review therefore pursues three objectives: (1) to document the ecological occurrence of *Pichia* across washed coffee fermentation systems; (2) to critically synthesize functional traits, including aroma production, enzymatic activity, stress tolerance, and bioprotective capacity, drawing on evidence from wine, beer, and other fermented beverages; and (3) to assess the practical potential and challenges of deploying *Pichia* species as starter cultures in washed coffee processing.

## 2 Occurrence of *Pichia* Among Native Coffee Fermentation Microbiota

Characterizing the occurrence of *Pichia* within the surrounding microbial community encompassing other yeasts, bacteria, and moulds provides critical insights into microbial ecology and informs predictions of *Pichia* behavior and its optimization as a starter culture in controlled fermentations. The surface of coffee cherries serves as the primary source of microbial inoculum during fermentation, with composition influenced by fruit maturity, climate, and agricultural practices (Vinícius de Melo Pereira et al. 2017). Surveys across diverse processing methods and producing regions indicate that yeast and bacterial taxa vary considerably between ecosystems, with more than 50 species implicated in coffee fermentation (de Melo Pereira et al. 2014; Elhalis, Cox, et al. 2023a; Haile and Kang 2019c; Pereira et al. 2024; Silva et al. 2013).

### 2.1 Traditional Washed Coffee Fermentation

In the wet process, commonly reported yeast species include *Pichia fermentans*, *P. kluyveri*, *Meyerozyma guilliermondii* (formerly *Pichia guilliermondii*, syn. *Candida guilliermondii*; hereafter *M. guilliermondii*), *Pichia kudriavzevii* (syn. *Issatchenkia orientalis*, *Candida krusei*; hereafter *P. kudriavzevii*), and *Meyerozyma caribbica* (formerly *Pichia caribbica*, syn. *Candida caribbica*; hereafter *M. caribbica*) alongside other yeasts such as *Candida railenensis*, *Candida glabrata*, *Candida quercitrusa*, *Cystofilobasidium* spp., and *Rhodotorula* spp., as well as lactic acid bacteria (LAB) including *Lactobacillus*, *Lactococcus*, and *Pediococcus*, have been reported during wet coffee fermentation (de Melo Pereira et al. 2014; Elhalis, Cox, and Zhao 2020; Evangelista et al. 2015). Evangelista et al. (2015) reported differential yeast populations between two farms, Lavras (L) and Monte Carmelo (MC), with counts ranging from 2.0 to 4.9 log CFU/g (Evangelista et al. 2015). *Torulaspora delbrueckii* was the predominant species at one location, exhibiting the highest initial load (5.3 log CFU/g), although its abundance fluctuated during fermentation. *M. caribbica* dominated at the other locations, with counts rising marginally from 4.4 to 4.9 log CFU/g. *Hanseniaspora uvarum* increased modestly from 3.7 to 3.8 log CFU/g after 36 h at one farm but exhibited limited presence at the other. de Melo Pereira et al. (2014) observed a substantial increase in yeast populations, from 2.7 log CFU/mL at the start of fermentation to a peak of 7.15 log CFU/mL after 40 h, before declining to 5.2 log CFU/mL at 48 h. *P. fermentans* was the most frequent isolate, followed by *P. kluyveri*, *C. glabrata*, and *C. quercitrusa*. *Saccharomyces* sp. appeared at 24 and 32 h, while *M. guilliermondii*, *M. caribbica*, and *Hanseniaspora opuntiae* were typically detected at the onset of fermentation. Consistent patterns were reported by Elhalis, Cox, and Zhao (2020), who identified six yeast species during coffee fermentation, with total populations rising from approximately 4 to 5.5 log CFU/g over 36 h. *H. uvarum* dominated, reaching 5.2 log CFU/g, followed by *P. kudriavzevii* at 4.6 log CFU/g. *C. railenensis* declined sharply from 3.6 to 1.5 log CFU/g, whereas *Candida xylopsoci* increased slightly from 2.0 to 2.5 log CFU/g. *P. fermentans* remained stable at low levels, while *Wickerhamomyces anomalus* (formerly *Pichia anomala*, syn. *Hansenula anomala*; hereafter *W. anomalus*) decreased marginally. Similarly, Ying et al. (2024) identified *P. kudriavzevii* and *Saccharomyces cerevisiae* as the predominant yeast species in the natural fermentation broth of Sidamo coffee beans (southern Ethiopia) during screening for indigenous beneficial isolates (Ying et al. 2024). These occurrence patterns indicate that *Pichia* are frequently associated with wet coffee fermentation ecosystems; however, their distribution is highly variable across studies, farms, and geographical regions. This variability suggests that species dominance is not intrinsic but environmentally driven, shaped by oxygen availability, acidification rate, nutrient composition, and fermentation management practices (Elhalis, Cox, et al. 2023a; Elhalis et al. 2021a; Haile and Kang 2019a, 2019b). *P. kudriavzevii* tends to persist in later fermentation stages due to its high tolerance to acid, osmotic, and thermal stress (Mukherjee et al. 2017; H. Xiao et al. 2014), whereas *P. kluyveri* and related strictly respiratory species are more transient due to their limited anaerobic adaptability (Battjes et al. 2024). These ecological differences indicate that *Pichia* species occupy distinct functional niches rather than forming a uniform functional group.

Collectively, these studies confirm that *Pichia* species are frequently reported in wet coffee fermentation, although their occurrence is inconsistent across locations. During wet coffee fermentation, exemplified by submerged fermentation (SmF), microorganisms are exposed to a dynamic and increasingly stressful environment (Elhalis, Cox, et al. 2023a; Elhalis et al. 2021a; Haile and Kang 2019c). Although SmF theoretically allows microbial growth in a liquid medium with improved control over environmental parameters compared with dry processing, such control is rarely achievable in coffee fermentation, which is typically conducted at a large scale in open systems. In practice, wet coffee fermentation presents multiple constraints that influence microbial growth and metabolite formation. Oxygen availability is often limited by restricted diffusion in the liquid matrix and is further reduced as microbial respiration intensifies, leading to a transition from aerobic to microaerophilic and anaerobic conditions. Simultaneously, pH decreases due to organic acid accumulation, while ethanol and other inhibitory metabolites increase, collectively imposing physiological stress. Limited mixing restricts nutrient and oxygen distribution, and the open nature of the system increases susceptibility to environmental contamination, which may alter microbial succession and fermentation outcomes. Consequently, microorganisms persisting under these conditions are capable of tolerating low oxygen availability, acidic pH, and metabolite accumulation while maintaining sufficient growth and metabolic activity to remain competitive within open fermentation systems (Elhalis, Cox, et al. 2023a; Elhalis et al. 2021a; Haile and Kang 2019c). The persistence and predominance of certain yeast species, particularly *Pichia* spp., under these challenging conditions reflects their tolerance to multiple stressors and adaptive capacity to sustain metabolic activity throughout fermentation, underscoring their potential as robust candidates for deliberate inoculation in controlled fermentations aimed at enhancing consistency, efficiency, and flavor development.

### 2.2 Modern Washed Coffee Fermentation Methods

In modern coffee processing, several strategies have been developed to modify the fermentation atmosphere, including self‐induced anaerobic fermentation and the use of CO_2_‐saturated, oxygen‐depleted environments. These approaches may be applied using either intact coffee cherries or depulped beans. Under such anaerobic conditions, sugars are metabolized by microorganisms through fermentative pathways, producing ethanol, CO_2_, glycerol, organic acids, and volatile compounds that enhance sweetness, acidity, and aroma complexity. Maintaining the beans within this controlled environment can further modulate endogenous metabolic activity, influencing final product quality relative to traditional open fermentations (Elhalis et al. 2021c; Kartika et al. 2024; Mauricio et al. 1998).

A similar SIAF approach was later applied under wet fermentation conditions, where mechanically depulped coffee beans were fermented in closed bioreactors containing ozonated water (da Silva Vale et al. 2023). Microbial profiling revealed a community dominated early by *Enterobacteriaceae* (mainly *Erwinia* and *Enterobacter*), which progressively declined as *Lactobacillaceae* (notably *Lactobacillus*) increased after 48. Yeasts, in contrast, remained subdominant throughout fermentation, with *Torulaspora* being the only genus exceeding 1% abundance, while *Pichia*, *Candida*, *Saccharomyces*, *Wickerhamomyces*, *Kazachstania*, and *Kluyveromyces* appeared at lower levels. Notably, *Pichia* spp. were detected at low abundance under these submerged conditions, suggesting that oxygen limitation, reduced mucilage contact, and altered mass transfer significantly constrain their ecological competitiveness. This contrasts with their higher prevalence in semi‐aerobic traditional wet fermentations, indicating that *Pichia* occupies an intermediate ecological niche requiring limited but not fully absent oxygen availability (Elhalis et al. 2021a; Elhalis, Cox, et al. 2023a; da Silva Vale et al. 2023). Across these SIAF‐based fermentations, altitude and fermentation matrix (whole cherry vs. depulped) strongly shape microbial ecology. *Pichia* abundance declined under submerged, water‐based fermentations (semi‐anaerobic, contrasting with the semi‐aerobic conditions typical of classic wet processes). This suggests that oxygen availability, mucilage contact, and related environmental variables modulate *Pichia* growth and metabolism, warranting further investigation. Importantly, isolates obtained under specific fermentation conditions should be evaluated for their stress tolerance and suitability. Such assessments are essential for selecting starter cultures tailored to particular fermentation methods, rather than assuming a single strain will perform optimally across all processing contexts, as discussed subsequently.

Few studies have investigated the microbial dynamics under carbonic maceration. Brioschi Junior et al. (2021) provided one of the earliest insights into the dynamics of microbial community changes during carbonic maceration. The study employed denaturing gradient gel electrophoresis (DGGE) and alpha diversity indices to characterize the microbial profile throughout fermentation. The findings revealed a clear link between microbial diversity and coffee quality. Specifically, higher bacterial diversity was associated with enhanced sensory attributes, suggesting that the composition of the microbial community critically determines the aroma, flavor, and overall quality of the beverage. Temperature and fermentation duration were also shown to shape microbial succession, with certain bacterial groups thriving under specific conditions and contributing to key sensory properties. For example, fermentation at 38°C for 96 h correlated with higher global sensory scores, indicating that controlled microbial activity can directly influence flavor development. These results prompted further investigation using next‐generation sequencing (NGS), which allows more precise identification of bacterial taxa contributing to both coffee quality and food safety. Entringer et al. (2024) evaluated fermentation at three temperatures (18°C, 28°C, and 38°C) and five durations (24, 48, 72, 96, and 120 h). The results indicated that fermentation temperature had a stronger influence on microbial composition than fermentation time. At 18°C, several taxa, including *Pichia cephalocereana*, *Hanseniaspora guilliermondii*, *Kurtzmaniella* sp., *Hanseniaspora nectarophila*, *M*. *guilliermondii*, *Cladosporium tenuissimum*, and members of the order *Chaetothyriales*, were consistently present at all fermentation times. At 28°C, *P*. *cephalocereana*, *H*. *guilliermondii*, *Kurtzmaniella* sp., *H*. *nectarophila*, *M*. *guilliermondii*, and *C*. *tenuissimum* persisted throughout fermentation, whereas at 38°C, no taxon was consistently present across all time points. Fungal relative abundance patterns also varied with temperature. *Pichia* dominated at 18°C and 28°C regardless of fermentation time, with *P*. *cephalocereana* exceeding 70% relative abundance. At 38°C and 120 h, however, *Diutina* and other genera increased in abundance, and *P*. *cephalocereana* was not detected. Other dominant genera across temperatures included *Fusarium*, *Ceratobasidium*, *Xylaria*, *Trichoderma*, *Kazachstania*, *Cladosporium*, *Wickerhamomyces*, *Debaryomyces*, *Candida*, and *Kluyveromyces*. The bacterial community dynamics and sensory quality of coffee were also studied by da Silva et al. (2023). Beta diversity was highest at 38°C, particularly between 96 and 120 h, indicating greater shifts in microbial composition at elevated temperatures. In contrast, the bacterial composition remained relatively stable at 18°C and 28°C, with *Leuconostoc* dominating throughout fermentation. At 38°C, notable succession occurred: *Weissella* increased after 48 h, and *Lactobacillus* predominated in the later stages (96–120 h), including *L*. *plantarum*, *L*. *brevis*, *L*. *fermentum*, *L*. *amylovorus*, and *L*. *reuteri*. Temperature also influenced microbial diversity and evenness: the highest richness was observed at 28°C after 24 h, while Pielou's evenness peaked at 38°C regardless of time. Sensory analysis revealed a significant improvement in coffee scores at 38°C, with increases strongly correlated with the relative abundance of *Lactobacillus* (*r* = 0.63, *p* = 0.03), whereas no significant correlations were observed at 28°C or 18°C. These results indicate that higher fermentation temperatures promote microbial succession that is directly linked to enhanced sensory quality, emphasizing the role of temperature in shaping both microbial ecology and the flavor profile of coffee. A thorough understanding of the changes occurring under these artificial atmospheric conditions is essential for guiding, optimizing, and predicting *Pichia* behavior during controlled fermentation processes. Table 1 shows the occurrence and characteristics of *Pichia* isolates in wet coffee fermentation methods.

**TABLE 1:** Occurrence and characteristics of *Pichia* isolates across wet coffee fermentation methods.

Method/process	*Pichia* isolate(s)	Occurrence/key characteristics	References
Wet/washed (SmF)	*Pichia fermentans*, *Pichia kluyveri*, *Meyerozyma guilliermondii*, *Meyerozyma caribbica*, *Pichia kudriavzevii*	Abundant at 2.7–7.15 log CFU/mL over 36–48 h; *P*. *fermentans* most frequent; survive low pH, low oxygen, increasing ethanol; early, mid, and late appearance varies by species	de Melo Pereira et al. (2014); Evangelista et al. (2015); Elhalis, Cox, and Zhao (2020); Ying et al. (2024)
SIAF/submerged with water (pilot/bioreactor)	*Wickerhamomyces*	Reduced *Pichia*/*Wickerhamomyces* abundance compared to dry/depulped SIAF; low oxygen and lower mucilage contact limit growth	da Silva Vale et al. (2023)
Carbonic maceration (anaerobic, CO_2_‐saturated)	*Pichia cephalocereana*, *M*. *guilliermondii*	*Pichia* dominance at 18°C–28°C across time points; suppressed at 38°C; abundance strongly temperature‐dependent	Entringer et al. (2024); Brioschi Junior et al. (2021); da Silva et al. (2023)

Abbreviations: CFU, colony‐forming units; RA, relative abundance.

## 3 Translating Basic Knowledge of *Pichia* Fermentation Activities to Coffee

The genus *Pichia* (*Saccharomycetaceae*) comprises approximately 100 species of yeast‐like fungi and has recently gained prominence as a starter or co‐starter culture in food and beverage fermentations due to its robustness and metabolic versatility. Several species, including *W*. *anomalus*, *P*. *kudriavzevii*, *P*. *fermentans*, *P*. *kluyveri*, and *Debaryomyces hansenii*, exhibit tolerance to heat, low pH, and fermentation inhibitors; produce a wide range of hydrolytic enzymes and aroma‐active metabolites; and participate in organic acid transformations that can positively influence product quality. Collectively, these traits position *Pichia* yeasts as promising candidates for modulating mucilage degradation, sugar metabolism, and volatile compound formation during coffee fermentation. The functional contributions of *Pichia* species across fermented beverage systems are primarily mediated through four interconnected biochemical mechanisms relevant to coffee fermentation: (i) biosynthesis of volatile and nonvolatile metabolites, including esters, higher alcohols, and organic acids; (ii) degradation of complex substrates through carbohydrate‐ and amino acid‐metabolizing pathways; (iii) extracellular hydrolytic activities, particularly pectinolytic and polysaccharide‐degrading enzymes involved in mucilage breakdown; and (iv) biocontrol mechanisms mediated by competitive exclusion, stress tolerance, and volatile organic compounds. Species‐ and strain‐dependent variation in the regulation and expression of these pathways is discussed in subsequent sections. Throughout this section, findings are categorized into three evidence tiers: (i) direct evidence from coffee fermentation systems; (ii) extrapolated evidence from analogous fermented food and beverage systems; and (iii) hypotheses requiring experimental validation in coffee contexts. This framework distinguishes established knowledge from informed inference. Direct evidence from coffee fermentation systems, however, remains limited due to the structural complexity of coffee matrices and the substantial variability among processing methods, which constrain controlled experimentation and reproducibility. Consequently, much of the current understanding of *Pichia* functionality in coffee must be inferred from studies conducted in other fermented food and beverage systems. Such studies provide valuable insights into *Pichia* growth behavior, metabolic activity, and stress tolerance across diverse environmental conditions. Integrating evidence from these systems enables a more robust evaluation of the potential functional relevance of *Pichia* yeasts in coffee fermentation, while also highlighting critical knowledge gaps. Addressing these gaps is essential to support the rational design, targeted application, and scalable implementation of *Pichia* yeasts in controlled coffee fermentation processes. Such efforts are expected to accelerate practical adoption, facilitate process optimization, and improve the predictability of *Pichia* behavior across diverse fermentation environments. Accordingly, the following sections synthesize current knowledge on commonly studied *Pichia* species, with particular emphasis on their fermentation behavior under aerobic and oxygen‐limited conditions and their selected applications in fermented products.

### 3.1 W. anomalus

#### 3.1.1 Fermented Beverages

Drawing on extrapolated evidence from wine, *W*. *anomalus* is a ubiquitous non‐*Saccharomyces* yeast commonly associated with grape, must, and winery environments. It frequently appears during the early stages of fermentation, even in musts inoculated with *S. cerevisiae*, and can tolerate ethanol levels up to 12.5% (v/v) while producing killer toxins that suppress competing yeasts (Heard and Fleet 1985; Padilla et al. 2018; Walker 2011). Ecological studies have identified it as a dominant species on grape surfaces, accounting for up to 25% of isolates from Grenache and Shiraz musts, and as the second most abundant yeast after *H*. *uvarum* in Cabernet Sauvignon (a red wine grape variety) (Bagheri et al. 2015; Cordero‐Bueso et al. 2011). Its metabolic and enzymatic capabilities reflect its physiological traits discussed above. At the biochemical level, its pronounced ester profile is mainly driven by a reversed esterase reaction, in which an esterase catalyzes ethanol and acetyl‐CoA condensation to form ethyl acetate (Rojas et al. 2002; Yoshioka and Hashimoto 1981). This metabolic distinction confers greater flexibility in ester production even when conventional AAT substrates are limited, as demonstrated by comparative fermentation studies showing that *W*. *anomalus* generated up to 4143 mg/L ethyl acetate after 48 h under aerobic conditions, compared to approximately 22 mg/L produced by *S. cerevisiae* under identical conditions (Rojas et al. 2001).

*W*. *anomalus* is recognized as a prolific producer of enzymes relevant to winemaking (Madrigal et al. 2013). Its glycosidase repertoire, encompassing β‐d‐glucosidase, α‐l‐arabinofuranosidase, α‐l‐rhamnosidase, and β‐d‐xylosidase, liberates bound volatile compounds from glycosylated precursors, directly enhancing aroma complexity. Several strains maintain enzymatic stability under high ethanol and acidic pH conditions typical of fermentation systems (Charoenchai et al. 1997; López et al. 2015; Manzanares et al. 1999; Mateo et al. 2011; Rosi et al. 1994; Spagna et al. 2002). Other enzymes, such as multifunctional exo‐β‐1,3‐glucanases, further enhance aroma while reducing wine viscosity (Gil et al. 2005; Sabel et al. 2014; Schwentke et al. 2014), while proteolytic enzymes have been explored as alternatives to bentonite for protein haze removal, offering a more sustainable clarification method that preserves flavor (Schlander et al. 2017). Regarding aroma, the species is notable for synthesizing acetate esters. Although traditionally regarded as a spoilage organism due to excessive ethyl acetate formation (> 150 mg/L), many strains produce balanced amounts of fruity esters such as isoamyl acetate and 2‐phenylethyl acetate that positively influence wine aroma (Domizio et al. 2011; Viana et al. 2008). These variations highlight the importance of strain selection when designing mixed fermentations. Importantly, *W*. *anomalus* demonstrated the ability to reduce or even eliminate undesirable compounds from wine, such as alkaloids, including putrescine and 4‐hydroxymandelonitrile. In a recent study on raspberry juice fermentation, *W*. *anomalus* improved the product's compositional profile and removal of toxic alkaloids such as putrescine while enhancing antioxidant capacity and α‐glucosidase inhibitory activity (Qin et al. 2024).

Another remarkable characteristic of *W*. *anomalus* is its ability to produce killer toxins with broad antimicrobial spectra (G.‐L. Liu et al. 2015; Passoth et al. 2011). Biocontrol capacity is further exerted through the production of phenylacetic acid, which suppresses competing spoilage fungi by inhibiting spore germination and mycelial development, as evidenced by significant reductions in colony diameter and spore germination rates under coculture conditions (Godana et al. 2020; M. Zhu et al. 2023). These proteins provide a natural biocontrol mechanism against spoilage yeasts, particularly Dekkera/Brettanomyces (Ciani and Comitini 2011; Comitini et al. 2004; Fernández de Ullivarri et al. 2018; Lopes et al. 2009). For example, the killer toxin Pikt was effective against multiple Brettanomyces isolates and remained stable in wine for at least 10 days (Comitini et al. 2004; Jessica et al. 2009; Oro et al. 2016). Other killer proteins, such as KTCf20, exhibit stability and activity under oenological conditions, binding to β‐1,3‐ and β‐1,6‐glucans on sensitive cell walls (Fernández de Ullivarri et al. 2018). Such antimicrobial properties position *W*. *anomalus* as a promising biocontrol agent and a potential alternative to sulfur dioxide in winemaking (Oro et al. 2016).

#### 3.1.2 Extending Lessons for *W. anomalus* to Coffee

**FIGURE 1:**
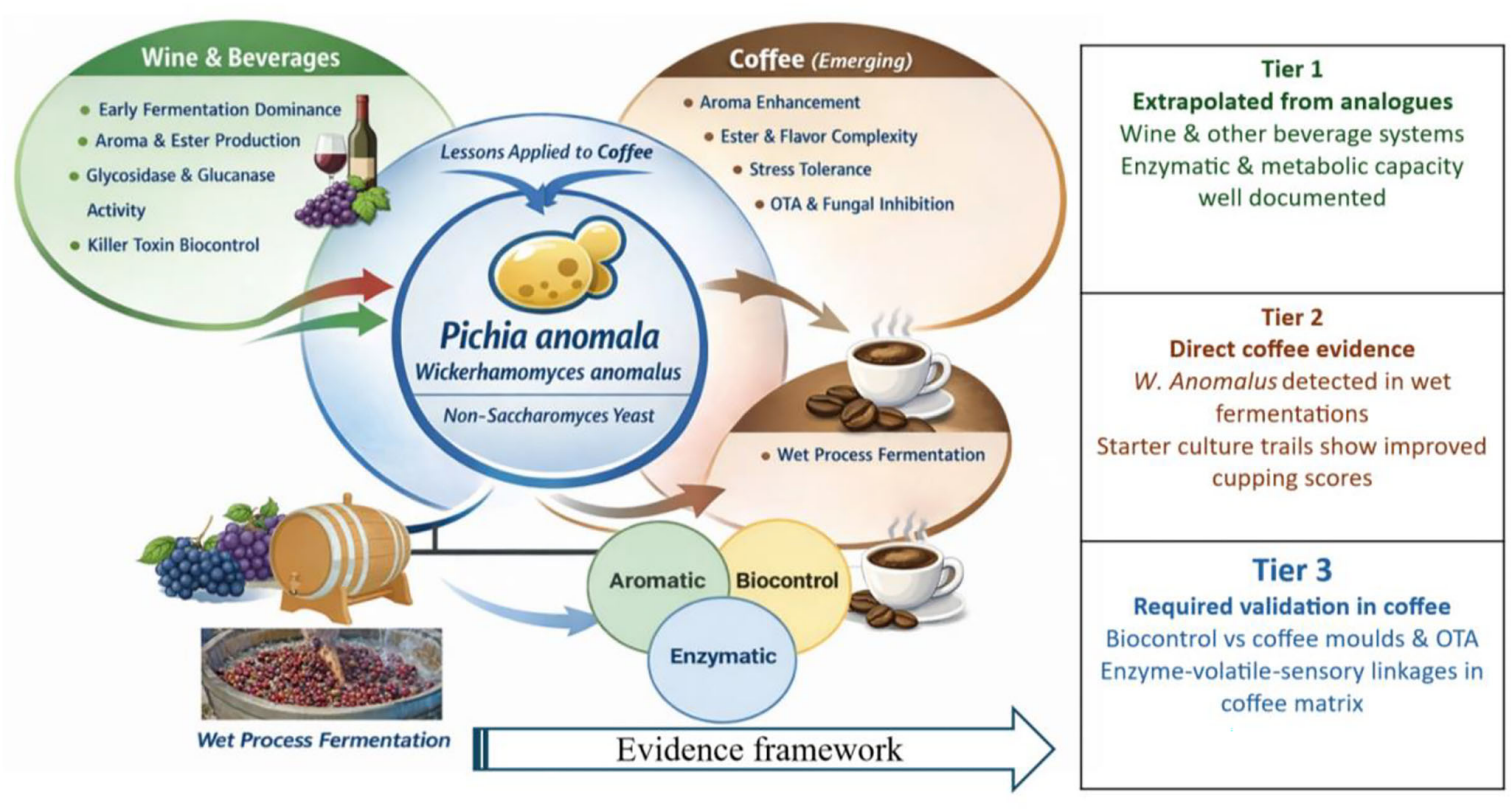
Multifunctional roles of *Wickerhamomyces anomalus* (*Pichia anomala*) in fermented beverages and potential applications in coffee.

*W*. *anomalus* represents a metabolically versatile and technologically valuable non‐*Saccharomyces* yeast. Its enzymatic activities, ester production, and biocontrol underline its dual role as both a beneficial contributor to sensory and a biological control agent, depending on strain characteristics and fermentation conditions. The insights gained from its use in fermented beverages provide an important foundation for understanding the behavior and potential of *W*. *anomalus* in other new‐era fermentation contexts, such as coffee processing. When translating these findings to coffee, however, it is essential to distinguish between what has been directly demonstrated in coffee systems, what is extrapolated from other fermented beverages, and what remains hypothetical (Figure 1; Table 2). Given the susceptibility of green coffee beans to fungal contamination and ochratoxin A (OTA) accumulation during drying and storage (López‐Rodríguez et al. 2024; Pardo et al. 2004), its demonstrated ability to inhibit fungal growth, sustain metabolic activity under stress, and generate desirable aroma compounds positions it as a compelling bioprotective and aroma‐enhancing agent in coffee bioprocessing. Collectively, these findings underscore the multifunctional capacity of yeast, linking its proven efficacy with emerging opportunities to improve the safety, quality, and sensory attributes of fermented coffee. Although *W*. *anomalus* has been consistently detected in wet coffee fermentation processes (Schwan et al. 2023), only limited research has focused on its deliberate application as a starter culture. This represents a major knowledge gap in coffee microbiology, particularly given its strong performance in model fermentation systems and its demonstrated enzymatic capacity to act on plant‐derived substrates. Haile and Kang (2020) inoculated 600 g of sterile, rehydrated green coffee beans (*Coffea arabica* L.) with *W*. *anomalus* at a concentration of 1.0 × 10^4^ CFU/g and fermented them at 30°C for 24 h (Haile and Kang 2020). The beans were subsequently washed and oven‐dried at 45°C until a moisture content of 10%–12% was achieved. Consumer sensory evaluation indicated that non‐fermented coffee was rated higher for color, sourness, mouthfeel, acidity, and overall quality, whereas fermented coffee was preferred for its aroma, bitterness, and astringency. Although no detailed chemical characterization was performed, fermentation markedly enhanced the functional properties of coffee, as evidenced by significantly higher antioxidant activity (DPPH and FRAP assays) and increased total phenolic content (TPC) and total flavonoid content (TFC) (*p* ≤ 0.05) across all roasting treatments (Haile and Kang 2020). These outcomes align with the proposed contribution of hydrolysis enzyme activity discussed earlier, which may enhance mineral bioavailability and antioxidant potential. In another study, Mahingsapun et al. (2022) evaluated the impact of *W*. *anomalus*, used alone and in combination with *P*. *kluyveri*, on the quality of wet‐processed Arabica coffee under varying fermentation conditions (10°C, 19°C, and 25°C; 14–18 h) (Mahingsapun et al. 2022). Coffees inoculated with *W*. *anomalus* achieved a cupping score of 79.7, compared with 75.6 in the uninoculated control, and were characterized by desirable sensory notes of nutty, spicy, perfume, rose, floral, caramel, roast, orange, and green apple. The highest cupping score (82.4) was obtained from beans fermented at 25°C for 10 h using a mixed inoculum of *P*. *kluyveri* and *W*. *anomalus*, which yielded complex sensory attributes, including stone fruit, sugarcane, banana, ripe fruit, orange, apple, red grape, brown sugar, tomato, berry, honey, citrus, and lemon. This optimized condition was subsequently scaled up to a 100 kg wet fermentation trial, in which depulped Arabica beans were inoculated with a 10% (v/w) seed culture and fermented in on‐site tanks at 10°C–15°C for 44 h. The scaled‐up process produced a cupping score of 77, compared to 73 for the control, with flavor notes of black tea, honey, lemon, and pronounced acidity. The control samples, in contrast, exhibited green, hay, roasted, and lime peel notes. Statistically significant improvements were recorded in several sensory parameters: fragrance/aroma, flavor, acidity, body, balance, sweetness, and overall quality. The authors attributed the slightly lower score at scale‐up compared with laboratory results to variables such as tank design, limited aeration (due to the absence of agitation), inoculum density, and temperature fluctuations (Mahingsapun et al. 2022). Further work by the same research group employed a UV‐induced mutant strain of *W*. *anomalus* in Arabica coffee fermentation (Meeampun et al. 2023). The mutant strain yielded a higher cupping score (82.0) than both the uninoculated control (75.5) and the wild‐type strain (80.25), producing distinct sensory attributes described by Q‐graders as pineapple, nutty, cocoa, caramel, honey, and lemon. Subsequent characterization revealed that these mutant strains exhibited enhanced pectinase activity (Meeampun et al. 2024). Consistently, Krajangsang et al. (2022) reported that fermentation with highly pectinolytic strains of *W*. *anomalus* resulted in superior cupping scores (80.25–84.75), whereas the control received the lowest evaluation (73.75). The control coffees were dominated by simple flavor notes such as caramel, vanilla, bell pepper, green vegetable, hay, and nutty tones, while inoculated samples developed more complex and desirable profiles characterized by floral, honey, prune, cocoa, and chocolate nuances (Krajangsang et al. 2022). While most studies report generally positive effects of *W*. *anomalus* on coffee quality, outcomes remain strongly dependent on strain, fermentation conditions, and process scale. Where sensory outcomes were less favorable—as with acidity and mouthfeel in Haile and Kang (2020)—context‐dependent process variables, including temperature, aeration, inoculum density, and microbial interactions, appear to govern performance rather than reflecting inherent strain limitations. A further limitation is the weak mechanistic linkage between enzymatic activity and volatile formation in coffee systems. Although glycosidase and pectinase activities are well established in wine and cereal fermentations as discussed above, their quantitative contribution in coffee remains largely unverified. As a result, enzyme–metabolite–sensory relationships in coffee fermentation are still predominantly inferred rather than experimentally demonstrated. Similarly, the biocontrol potential of *W*. *anomalus* killer toxins against coffee‐relevant spoilage fungi and mycotoxin‐producing moulds—while a plausible and well‐motivated hypothesis drawn from wine and post‐harvest systems—has not yet been directly tested in coffee matrices. Collectively, these findings underscore the promising role of *W*. *anomalus* as a starter culture across the early trials in coffee. Evidence from analogous systems demonstrates its capacity to adapt across diverse fermentation environments and, through enzymatic activity and aromatic metabolite production, to enhance both the sensory complexity and functional quality of fermented coffee.

**TABLE 2:** Roles of *Wickerhamomyces anomalus* in fermented foods and beverages, with implications for coffee fermentation.

Food/beverage	Functional roles/key contributions	Key metabolites/compounds	Sensory/quality impact	References	Lessons/implications/notes
Wine	Early fermentation yeast; survives up to 12.5% ethanol; biocontrol; enzyme producer (glycosidases, proteases, glucanases)	Acetate esters (isoamyl acetate, 2‐phenylethyl acetate); killer toxins (Pikt, KTCf20)	Enhances fruity and floral aroma; reduces viscosity; natural biocontrol; reduces undesirable alkaloids	Bagheri et al. (2015); Domizio et al. (2011); Heard and Fleet (1985); Padilla et al. (2018); Walker (2011)	Aroma enhancement, microbial control, enzymatic modulation, and strain selection strategies can guide starter culture design in coffee, confirms resilience in SmF
Raspberry juice	Fermentation starter, improves compositional profile	Alkaloid reduction (putrescine); antioxidant and α‐glucosidase activity	Improved functional quality	Qin et al. (2024)	Demonstrates metabolic resilience and functional metabolite production, relevant for coffee antioxidant and bioactive compound enhancement
Coffee	Detected in various coffee processes; produces esters, antioxidants; pectinolytic and phytase activities improve bioavailability and aroma	Esters (various); phenolics, flavonoids; enzymes (pectinases, phytases)	Enhances aroma complexity (nutty, floral, stone fruit, honey, citrus); improves functional quality; increases cupping scores	Haile and Kang (2020); Krajangsang et al. (2022); López‐Rodríguez et al. (2024); Mahingsapun et al. (2022); Meeampun et al. (2023); Pardo et al. (2004)	Cross‐domain lessons applied: optimize strain selection, fermentation temperature/time, inoculum density, co‐inoculation, mutant strains for enzyme activity; improve sensory and functional properties while controlling spoilage

### 3.2 P. kudriavzevii

#### 3.2.1 Fermented Beverages

In the beverage industry, *P*. *kudriavzevii* has attracted considerable interest due to its capacity to metabolize organic acids and flavor‐active compounds and secrete hydrolytic enzymes (Chu et al. 2023). *P*. *kudriavzevii* plays an increasingly recognized role in modulating wine and cider pH and malic acid content. It can reduce malic acid by up to 40% during alcoholic fermentation, increasing pH by approximately 0.3 units (del Mónaco et al. 2014, 2016). Malic acid is microbiologically unstable and must be removed before bottling to prevent unwanted secondary fermentations, especially in red wines. Traditionally, malic acid is degraded through malolactic fermentation by LAB; however, this process can cause quality issues, particularly in warm viticulture regions, such as color loss or the formation of undesirable compounds such as biogenic amines and ethyl carbamate. Consequently, there has been growing interest in alternative yeast species, including *P*. *kudriavzevii*, that can efficiently degrade malic acid during alcoholic fermentation (S. Benito 2020; Mazzucco et al. 2024, Mazzucco et al. 2025). At the biochemical level, *P*. *kudriavzevii* modulates fermentation aroma profiles through coordinated regulation of multiple metabolic pathways. It reduces higher alcohols such as isoamyl alcohol, isobutanol, and phenylethyl alcohol through downregulation of genes associated with the Ehrlich and Harris pathways while simultaneously enhancing ester synthesis, including ethyl butyrate, isoamyl acetate, and phenylethyl acetate, and volatile fatty acid production, encompassing hexanoic, octanoic, and nonanoic acids (Y.‐W. Wang et al. 2024). Transcriptomic evidence indicates that these effects are mediated by coordinated upregulation of glycolytic and ester biosynthesis genes, alongside activation of alcohol and acid metabolism pathways (Y.‐W. Wang et al. 2024). Metabolomic analyses further revealed concurrent depletion of sugars and amino acids, accompanied by increased levels of NAD^+^, NADH, ATP, and acyltransferases, reflecting a metabolic shift from classical glycolysis toward pathways favoring alcohol and aroma compound biosynthesis (D. Xu et al. 2019). Correlation analyses link 3‐methyl‐1‐butanol formation to leucine consumption via the Ehrlich pathway, 2‐phenylethanol production to shikimate pathway activity, and overall volatile accumulation to fatty acid metabolism intermediates (D. Xu et al. 2019; Y. Xu, Xu, et al. 2024; Y. Xu, Zhao, et al. 2024).

Beyond acidity management, wine fermentation provides a well‐controlled model system for elucidating yeast behavior, metabolic activity, and flavor modulation mechanisms transferable to more complex matrices such as coffee fermentation. Within this framework, Y.‐W. Wang et al. (2024) investigated mono‐ and coculture fermentations of *P*. *kudriavzevii* and *S. cerevisiae* under controlled kiwifruit wine fermentation conditions (28°C for 7 days). Compared with *S. cerevisiae*, *P*. *kudriavzevii* exhibited slower sugar utilization and ethanol production, reaching 9.08% (v/v) ethanol after 7 days, whereas *S. cerevisiae* reached 11.86% (v/v), despite comparable growth trajectories. Importantly, *P*. *kudriavzevii* produced substantially higher levels of total organic acids (42.04 mL), dominated by quinic, lactic, and malic acids. Although *P*. *kudriavzevii* biomass declined over the course of fermentation in the coculture, its metabolic influence persisted, demonstrating that population dominance is not necessarily required for flavor impact. Specifically, sustained metabolic effects included reduced formation of higher alcohols (isoamyl alcohol, isobutanol, and phenylethyl alcohol) and enhanced production of key aroma‐active compounds such as esters (ethyl butyrate, isoamyl acetate, and phenylethyl acetate) and volatile fatty acids (hexanoic, octanoic, and nonanoic acids). Supporting this observation, comparative analyses using headspace solid‐phase microextraction coupled with gas chromatography‒mass spectrometry (HS–SPME–GC‒MS) revealed that *P*. *kudriavzevii* produced approximately 3.5‐fold higher ester concentrations than *S. cerevisiae* during sorghum juice fermentation (J. Xiao et al. 2023), underscoring its capacity for ester‐driven aroma enhancement across substrates. Further evidence of the functional importance of *P*. *kudriavzevii* derives from apricot wine fermentation studies, which also serve as effective model systems for dissecting non‐*Saccharomyces* yeast behavior (Chen et al. 2023). Multivariate analyses and structural equation modeling identified *Pichia* as a keystone genus strongly associated with ester compounds (e.g., ethyl caprylate, ethyl caprate, isoamyl acetate), floral volatiles such as linalool, and reduced green‐note compounds (decanal and 1‐hexanol). Notably, yeast populations exerted a stronger direct influence on aroma profiles than *S. cerevisiae* alone, challenging the traditional dominance‐based paradigm of wine fermentation. Pronounced strain‐level differences were observed, with one *P*. *kudriavzevii* strain exhibiting high ethanol tolerance, completing fermentation independently, and producing wines with enhanced fruity and floral sensory attributes, whereas other strains negatively affected fermentation performance.

Regarding hydrolytic enzyme activity, a *P*. *kudriavzevii* strain isolated from the Helan Mountains produces intracellular β‐glucosidase with high glucose tolerance that is capable of hydrolyzing glycosidic precursors in grape must to release aroma‐active compounds. In controlled fermentations, Cabernet Sauvignon must was co‐inoculated with *S. cerevisiae* and β‐glucosidase, and fermentation was carried out statically at 25°C until residual sugars fell below 4 g/L (Yu et al. 2025). Aroma analysis quantified odor‐active compounds (OAV ≥ 1), grouped into terpenes, higher alcohols, esters, fatty acids, and carbonyl compounds. The addition of β‐glucosidase significantly increased the total terpenes from 0.36 mg/L in the control to 0.68 mg/L, including linalool (0.12 mg/L) and citronellol (0.09 mg/L), and the unique detection of caryophyllene (0.08 mg/L). Higher alcohols, including isoamyl alcohol (192.5 mg/L) and benzene alcohol (75.3 mg/L), were markedly elevated compared with the control (204.1 mg/L total higher alcohols). Odor‐active esters, such as ethyl caproate (12.6 mg/L), ethyl octanoate (15.4 mg/L), and isoamyl acetate (8.7 mg/L), increased substantially from control levels (37.5 mg/L total esters), enhancing floral and fruity notes while reducing green off‐flavors (Yu et al. 2025). These results illustrate the substrate specificity and functional impact of *P*. *kudriavzevii* β‐glucosidase on wine aroma composition, highlighting its potential as a functional yeast for targeted aroma enhancement, although further research is needed to optimize activity under practical winemaking conditions. Collectively, these findings demonstrate that *P*. *kudriavzevii* can disproportionately shape fermentation chemistry through strain‐dependent metabolic specialization, providing a mechanistic framework—based on extrapolated evidence—potentially applicable to coffee processing, where analogous substrates and fermentation dynamics may support similar functional roles.

#### 3.2.2 Extending Lessons for *P. kudriavzevii* to Coffee

**FIGURE 2:**
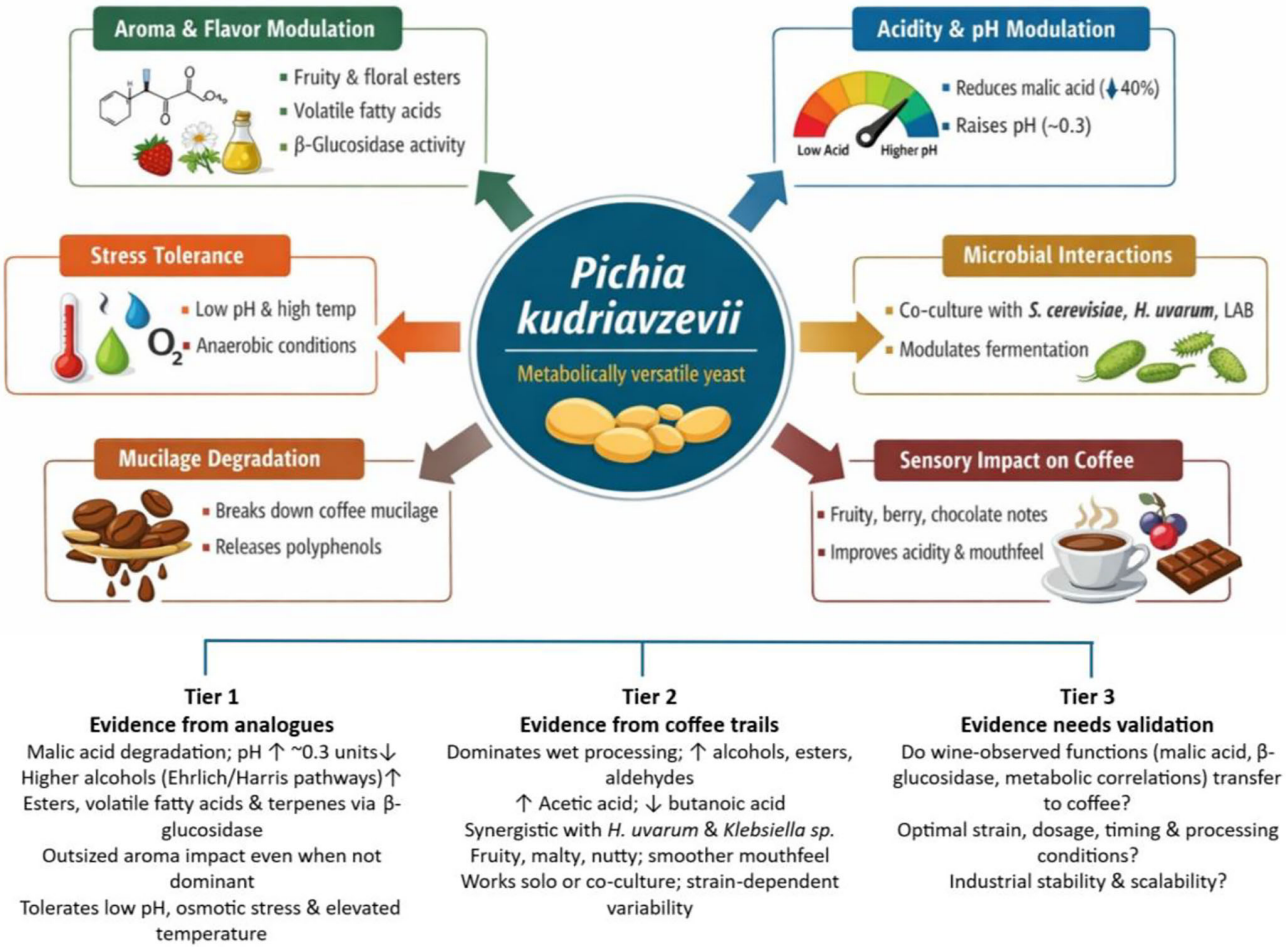
Fermentation potential of *Pichia kudriavzevii*, cross evidence from fermented beverages and potential applications in coffee.

When translating these beverage‐derived findings to coffee, it is important to distinguish between what has been directly demonstrated in coffee systems, what is extrapolated from other fermented beverages, and what remains hypothetical (Figure 2). *P*. *kudriavzevii* has been shown to act as a key modulator of flavor development. Its ecological success in fermentation systems is strongly linked to its physiological robustness, particularly tolerance to low pH, high sugar concentrations, osmotic stress, and elevated temperatures (Akita and Matsushika 2023; Mukherjee et al. 2017), enabling it to persist in early fermentation stages where many non‐*Saccharomyces* yeasts decline. By extrapolation from wine and fruit fermentation systems, correlation analyses demonstrate strong associations between the presence of *P*. *kudriavzevii*, even under suppressed growth, and the production of aroma‐active esters, including ethyl acetate, isoamyl acetate, and phenethyl acetate, alongside hydrolytic enzyme activity and acid modulation (Table 3). These associations represent extrapolated evidence; whether equivalent metabolic relationships operate under the distinct substrate and processing conditions of coffee fermentation remains to be directly verified. Such metabolic traits, robust stress tolerance, efficient sugar metabolism, and high volatile production capacity suggest that *P*. *kudriavzevii* could survive, influence the fermentation process, and potentially play a strategic role in modulating key flavor compounds while supporting reproducible sensory profiles under the complex and dynamic conditions of coffee fermentation.

**TABLE 3:** Roles of *Pichia kudriavzevii* in fermented foods and beverages, with implications for coffee fermentation.

Food/beverage	Functional roles/key contributions	Key metabolites/compounds	Sensory/quality impact	References	Lessons/implications/notes
Wine/cider	Reduces malic acid up to 40%, increasing pH ∼0.3; alternative to malolactic fermentation; secretes hydrolytic enzymes and flavor‐active compounds	Organic acids; flavor‐active esters; β‐glucosidases	Modulates acidity and pH; prevents unwanted secondary fermentation; enhances aroma complexity	Bautista‐Ortín et al. (2013); S. Benito (2020); Chu et al. (2023); del Mónaco et al. (2014, 2016); Mazzucco et al. (2024, 2025); Yu et al. (2025)	Potential to modulate coffee bean acidity, support aroma development, increase antioxidant content, and confirms resilience in SmF
Sorghum juice fermentation	Produces higher ester content than *Saccharomyces cerevisiae*; enhances aroma formation	Esters (3.5× more than *S. cerevisiae*)	Fruity aroma enhancement	J. Xiao et al. (2023)	Highlights potential for boosting ester‐related fruity/floral notes in coffee fermentation
Coffee	Starter candidate for semidry, wet, and anaerobic fermentations; dominates microbial communities; produces alcohols, aldehydes, esters; modulates pH and sugar metabolism; compatible with other functional microbes	Alcohols (ethanol, isoamyl alcohol, phenylethyl alcohol); aldehydes (acetaldehyde, 3‐methylbutanal); esters (ethyl acetate, isoamyl acetate, phenethyl acetate); polyphenols, chlorogenic acids	Enhanced fruity, floral, chocolate, honey, berry, and nutty notes; improved balance, mouthfeel, acidity, and overall cupping scores	Aswathi et al. (2022); Elhalis, Cox, et al. (2023b); Elhalis et al. (2021b); Mariyam et al. (2022); Shankar et al. (2022); Taufik et al. (2024)	Enhances flavor via volatile metabolites and aroma release; increases antioxidants through pectinase‐driven mucilage degradation

Abbreviations: CFU, colony‐forming units; pH, acidity; TCA, tricarboxylic acid cycle; VOC, volatile organic compounds.

Direct evidence from coffee confirms that *P*. *kudriavzevii* has emerged as a promising functional yeast, with demonstrated contributions to both biochemical transformation and sensory enhancement across diverse processing conditions. In wet‐process fermentation, *P*. *kudriavzevii* not only thrived but also dominated the microbial community, confirming its robustness (Elhalis et al. 2021b). Inoculated beans exhibited markedly higher concentrations of alcohols (ethanol, isoamyl alcohol, phenylethyl alcohol) and aldehydes (acetaldehyde, 3‐methylbutanal), correlating with enhanced aroma complexity. Sensory evaluation revealed contributions to malty, vanilla, and nutty notes, with higher overall flavor and acidity scores compared to controls. Mixed inoculation with *H*. *uvarum* demonstrated strong compatibility, with both yeasts thriving without competitive inhibition. The combined treatment amplified volatile production compared to single‐strain fermentations: isoamyl alcohol increased threefold with *H*. *uvarum*, fourfold with *P*. *kudriavzevii*, and nearly fourfold in the coculture; phenylethyl alcohol increased twofold, fivefold, and sixfold, respectively. Furfuryl alcohol and esters (ethyl and methyl acetate) were highest in the combined inoculation, and 3‐methylbutanal sharply increased after roasting, particularly in the mixed culture. Organic acid shifts included slight acetic acid enrichment and reduced butanoic acid in inoculated fermentations. These metabolic differences translated into superior sensory profiles, with the combined inoculation scoring highest overall (82.0), surpassing *P*. *kudriavzevii* alone (81.5), *H*. *uvarum* (79.8), and the control (80.3), featuring distinctive earthy, apple cider, and walnut notes with a smooth mouthfeel (Elhalis et al. 2021b). Mariyam et al. (2022) compared coffee fermentation under anaerobic and aerobic conditions, with or without yeast inoculation, to evaluate the functional role of *P*. *kudriavzevii*. Under anaerobic conditions, *P*. *kudriavzevii* maintained a stable fermentation temperature (29°C–31°C) and promoted moderate moisture uptake. Among inoculated treatments, *P*. *kudriavzevii* exhibited a consistent pH decline that was more gradual and sustained than *S. cerevisiae*, which partially rebounded after 48 h due to over‐fermentation. The study did not report mucilage degradation or sensory quality outcomes; the stronger acidification under anaerobic conditions reflected active sugar metabolism and organic acid formation, although its specific contribution to flavor precursor development in coffee remains to be directly demonstrated (Elhalis, Cox, et al. 2023a; Elhalis, Cox, Frank, et al. 2020; Elhalis et al. 2021b). Co‐inoculation of *P*. *kudriavzevii* with *Klebsiella sp*. further demonstrated that deliberate microbial pairing could enhance coffee flavor development (Taufik et al. 2024). At a 1:10 bacteria‐to‐yeast ratio with 15% inoculum (v/w), both bacterial and yeast populations increased by ∼2–3 log CFU/g compared to uninoculated controls after the first day, indicating competitive dominance over native microbiota. While *Klebsiella* dominated early fermentation due to rapid growth, only *P*. *kudriavzevii* persisted after drying, reflecting its tolerance to low pH and reduced water activity. Metabolically, *P*. *kudriavzevii* contributed to elevated levels of ethanol, acetaldehyde, ethyl acetate, isoamyl acetate, diacetyl, and acetoin, imparting fruity, buttery, and wine‐like sensory attributes. These changes correlated with higher cupping scores, particularly at the 15% inoculum level, with chocolate, ripe cherry, and tannin‐like flavors reminiscent of red wine. In contrast, imbalanced inoculation ratios were detrimental: excessive yeast (1:20) induced off‐flavors and bitterness, while low yeast (1:5) limited flavor complexity (Taufik et al. 2024). This evidence underscores the importance of controlled microbial ratios and inoculum sizes to consistently achieve specialty coffee profiles, positioning *P*. *kudriavzevii*, alone or in consortia, as a key functional yeast in coffee bioprocessing. Collectively, although improvements in sensory quality are frequently reported, the mechanistic relationship between enzymatic activity, microbial metabolism, and volatile compound formation in coffee systems remains largely inferential. Whether enzyme–metabolite–sensory relationships, acid modulation capacity, and hydrolytic enzyme contributions well characterized in wine and fruit systems operate equivalently in coffee matrices remains to be demonstrated. Contradictions across studies suggest that fermentation outcomes are strongly strain‐, environment‐, and process‐dependent rather than universally beneficial. Future studies should therefore focus on controlled comparative fermentations, strain‐level characterization, and integrated metabolomic and transcriptomic approaches to better define the ecological and biochemical role of *P*. *kudriavzevii* in coffee processing and support industrial standardization.

### 3.3 P. kluyveri

#### 3.3.1 Fermented Beverages

In alcoholic beverages, the primary contribution of *P*. *kluyveri* lies in aroma modulation rather than complete sugar fermentation or ethanol yield. Unlike *S. cerevisiae*, which metabolizes glucose, fructose, sucrose, and maltose, *P*. *kluyveri* predominantly consumes glucose, leaving other sugars largely unfermented (Huang et al. 2024; Zapryanova et al. 2025). This restricted carbohydrate utilization limits ethanol production (∼3.2% v/v) but allows survival in moderate ethanol concentrations (4%–5% v/v), making the yeast particularly suitable for low‐ or nonalcoholic beverages. *P*. *kluyveri* exhibits a remarkable ability to release thiol‐derived esters, notably 3‐sulfanylhexan‐1‐ol acetate (3‐SHA), which imparts passionfruit‐ and box tree‐like aromas highly valued in wine (Anfang et al. 2009). Sequential fermentations with *S. cerevisiae* further enhance these compounds, increasing 3‐SHA by 10%–72% and 3‐SHA by ∼40%, particularly when *P*. *kluyveri* dominates early in fermentation (1:9 inoculation ratio). These thiol‐derived esters are produced through alcohol acetyltransferase (AAT)‐mediated esterification of higher alcohols with acetyl‐CoA, with higher alcohols themselves originating from α‐ketoacid intermediates generated via both central carbon metabolism and the Ehrlich pathway, directly linking amino acid catabolism to volatile ester biosynthesis (Dzialo et al. 2017; Z. Zhu et al. 2024). Strain compatibility is critical, with optimal aroma enhancement observed when *S. cerevisiae* partners possess high β‐lyase activity (Anfang et al. 2009; Belda et al. 2016). Beyond thiol esters, *P*. *kluyveri* modulates higher alcohol levels, generally reducing total higher alcohols by ∼15%, which mitigates harsh sensory notes, although compound‐specific effects vary (hexanol—50%; 2‐phenylethanol—20%) (S. Benito 2019; S. Benito et al. 2015). While some studies report higher alcohols up to 25%, overall concentrations remain below the 350 mg/L threshold associated with varietal aroma masking, favoring clearer expression of varietal character (Dutraive et al. 2019; Ruiz et al. 2019). Genome sequencing has uncovered expanded gene families in *P*. *kluyveri*, including ADH6 and ADH7 encoding NADPH‐dependent alcohol dehydrogenases and LEU4 encoding α‐isopropylmalate synthase—absent in *S. cerevisiae*—with high expression of certain isoforms directly linked to elevated production of fusel alcohols and their acetate derivatives (Wei et al. 2022). Transcriptomic profiling further revealed stage‐specific expression of key Ehrlich pathway genes, including BAT1, ARO8, and decarboxylases PDC and ARO10, indicating rapid transformation of amino acids such as leucine and phenylalanine into higher alcohols and esters. Notably, despite lacking canonical ATF1 and EEB1 homologs—the primary AATs responsible for acetate ester formation in *S. cerevisiae*—*P*. *kluyveri* maintains robust acetate ester synthesis through alternative species‐specific acyltransferases, enabling HAA accumulation at concentrations two‐ to threefold higher than those observed in *S. cerevisiae* fermentations (Wei et al. 2022).

In tequila fermentations at 14°Brix (∼120 g/L sugar), *P*. *kluyveri* achieved high ethanol yield (*Y*_p/s_ > 0.45) and produced greater amounts of ethyl lactate than *S. cerevisiae* or *Kluyveromyces marxianus*, despite transient early accumulation of acetaldehyde (Amaya‐Delgado et al. 2013). Its inability to metabolize maltose or maltotriose has been exploited in nonalcoholic beer, yielding minimal ethanol (∼0.2% v/v) while significantly enhancing fruity esters, including ethyl acetate, isoamyl acetate, ethyl butyrate, ethyl hexanoate, and ethyl octanoate, producing beverages with greater aroma complexity than conventional nonalcoholic beers (SaerensJan and Swiegers 2014). In beer fermentations, *P*. *kluyveri* monocultures exhibit slow initial fermentation with minimal CO_2_ release and pH change during the first 3 days, followed by accelerated fermentation upon *S. cerevisiae* inoculation, completing in ∼9 days (∼2.1% v/v ethanol). Co‐fermentation markedly improved aromatic profiles: isoamyl acetate and 2‐phenethyl acetate increased ∼12‐fold, linalool by 1.17‐fold, and other ethyl esters, including ethyl laurate, increased up to 29‐fold. Simultaneously, higher alcohols such as 3‐methyl‐1‐butanol and 2‐phenylethanol decreased by up to 91.9%, producing more balanced, less harsh flavors. Sensory evaluation confirmed higher scores for intensity (3.76), balance (3.90), and fruitiness (3.95) compared to *S. cerevisiae* alone (3.14, 3.33, and 2.71, respectively) (Rong et al. 2025).

In sequential and co‐inoculated fermentations, the timing of inoculation and strain compatibility strongly influence *P*. *kluyveri*'s functional contribution. In low‐alcohol beer, coculture at a 1:10 ratio with *S. cerevisiae* markedly enhances aroma complexity without increasing ethanol content, whereas monocultures of *P*. *kluyveri* produce beers with poor sensory quality, characterized by high bitterness, weak carbonation, and unbalanced flavor (Huang et al. 2024). Mechanistically, *P*. *kluyveri* contributes disproportionately to the production of acetate esters such as isoamyl acetate and phenethyl acetate, generating fruity and floral notes, while *S. cerevisiae* ensures efficient sugar utilization, ethanol formation, carbonation, and foam stability. Similar dynamics have been observed in wine: co‐fermentation at a 9:1 ratio with *S. cerevisiae* delayed overall alcoholic fermentation by ∼3 days, with *P*. *kluyveri* persisting for up to 9 days at 14°C (Anfang et al. 2009). Sequential inoculation extended its activity for 6 days post‐*S. cerevisiae* addition (Beckner Whitener et al. 2016), whereas in some systems, a rapid decline occurred once *S. cerevisiae* dominated (S. Benito et al. 2015). The behavior of P. *kluyveri* in kombucha further illustrates the importance of microbial synergy in mixed‐culture fermentations, where interactions with other microorganisms, not yeast alone, determine metabolic outcomes, a concept directly relevant to coffee processing. When grown alone on sucrose, *P*. *kluyveri* exhibits minimal growth; however, co‐fermentation with a symbiotic culture of bacteria and yeast (SCOBY), a complex community of yeasts and bacteria used to ferment sweetened tea, enables efficient metabolism of glucose and fructose through invertase activity. This results in rapid acetic acid accumulation (≈2.5 g/L after 4 days) and the formation of aroma‐active acetate esters such as isoamyl acetate and 2‐phenethyl acetate, enhancing beverage fruitiness (van Wyk et al. 2023). Ethanol levels remain low due to consumption by acetic acid bacteria, and the TPC is largely unaffected, indicating that *P*. *kluyveri* primarily influences sugar metabolism and volatile compound formation rather than phenolic transformations. Collectively, these findings underscore that without synergistic partners, the growth and aroma production of *P*. *kluyveri* are constrained and that harnessing such microbial dynamics is essential to achieve reproducible flavor profiles under complex fermentation conditions. These beverage‐derived findings collectively provide an extrapolated mechanistic context for interpreting *P*. *kluyveri*'s behavior in coffee fermentation systems.

#### 3.3.2 Extending Lessons for *P. kluyveri* to Coffee

**FIGURE 3:**
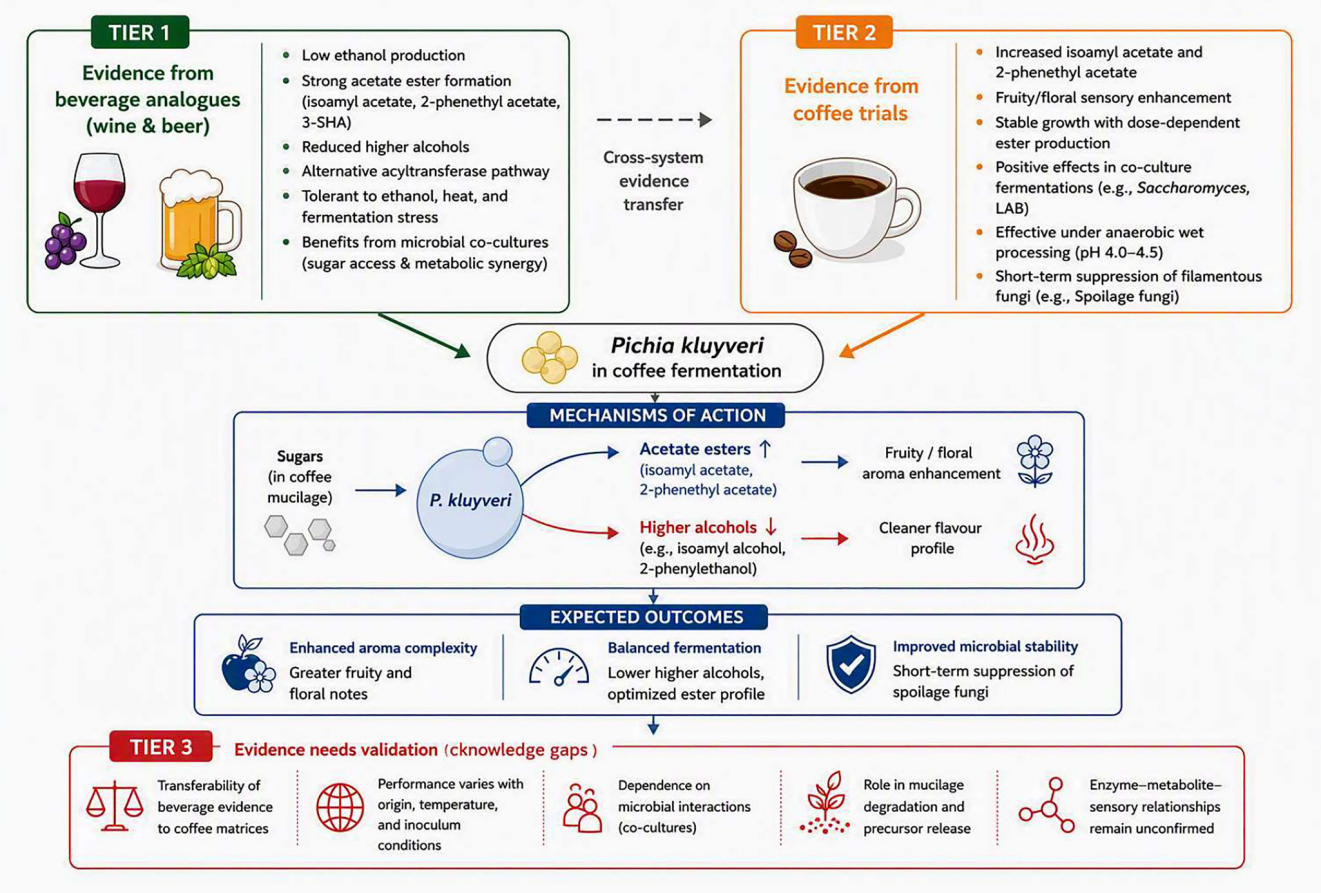
Aroma modulation and fermentation potential of *Pichia kluyveri* in fermented beverages.

*P*. *kluyveri* is strictly respiratory, with moderate ethanol production and a remarkable capacity for aroma modulation via acetate esters and higher alcohols. By extrapolation from wine, kombucha, and beer, its tolerance to ethanol, heat, and fermentation stresses, alongside its ester‐producing capacity, positions it as a secondary or auxiliary fermenter whose role is metabolic modulation rather than sugar depletion (Figure 3). However, its functional potential is highly dependent on microbial interactions, a dependency that has been directly observed in coffee systems as well. Direct evidence from coffee confirms that sterile green coffee beans inoculated with *P*. *kluyveri* (∼5.6 log CFU/mL) under wet‐processing conditions at 20°C for 6 days demonstrated strong flavor modulation potential through fruity ester production (C. Wang et al. 2020b). The yeast exhibited steady growth (∼2 log CFU/mL in the first 3 days) with minimal acidification (pH 5.8 → 5.7) and was uniquely associated with isoamyl acetate production. This resulted in a 2.4‐fold increase in 2‐phenylethyl acetate compared to uninoculated controls, imparting pronounced fruity and floral notes. In comparison, *S. cerevisiae* under the same conditions enhanced 2‐phenylethyl acetate by only 1.2‐fold and contributed more to organic acids and diacetyl, enhancing roasted and nutty attributes rather than fruitiness (C. Wang et al. 2020b). These findings are consistent with beverage fermentations, where *P*. *kluyveri* disproportionately enhances acetate ester synthesis despite relatively modest fermentative performance (Wei et al. 2022). Direct evidence further shows that Arabica beans inoculated with *P*. *kluyveri* at 19°C for 14 h increased yeast populations from 4 to 6 log CFU/mL, while pH decreased from 6.87 to 4.37, demonstrating strong adaptation and growth (Mahingsapun et al. 2022). Sensory evaluation revealed distinctive flavors, including peanuts, brown sugar, chocolate, smoke, spices, roasted notes, lemon, orange, green apple, and caramel, with cupping scores of 78.6, higher than uninoculated controls (75.6). Adjusting the fermentation temperature to 10°C–25°C further improved the scores to 80–81. This temperature‐dependent response suggests that environmental conditions strongly influence the metabolic expression and sensory contribution of *P*. *kluyveri* during coffee fermentation. The inoculum size of *P*. *kluyveri* significantly affects flavor development. Inoculation ranging from 10^5^ to 10^10^ CFU/g enhanced isoamyl acetate levels, with roasted beans showing ∼43% higher content than naturally fermented beans and coffee brewed from beans inoculated with 5 × 10^7^ CFU/g displaying ∼60% higher levels (Saerens and Swiegers 2014). These results demonstrate a dose‐dependent enhancement of aroma compounds, suggesting that even though *Pichia* is naturally present in coffee fermentations, additional inoculation can further boost ester production.

Mixed‐culture fermentations highlight *P*. *kluyveri*'s reliance on microbial synergy to overcome metabolic limitations, and similar interactions have been extensively reported in wine and other fermentations. Co‐inoculation with *S. cerevisiae* at a 10:1 ratio (5.6 vs. 4.6 log CFU/mL) enabled steady growth of *P*. *kluyveri* to 8.1 log CFU/mL over 6 days, a marked improvement over monoculture fermentation (C. Wang et al. 2020a). This enhancement is attributed to sucrose hydrolysis by *S. cerevisiae*, releasing glucose and fructose as accessible carbon sources for *P*. *kluyveri*, which alone cannot efficiently utilize sucrose. Coculture elevated isoamyl acetate and 2‐phenylethyl acetate by 1.2‐ and 4.1‐fold, imparting fruity and winey aromas that partially survived roasting. Sequential pre‐fermentation with *Lactococcus lactis* subsp. *cremoris* further promoted ester formation by modulating medium acidity and generating additional reactive sugars, enhancing Maillard and caramelization reactions during roasting. While *S. cerevisiae* primarily contributed to organic acids and diacetyl (nutty and roasted notes), *P*. *kluyveri* drove fruity complexity through acetate ester synthesis from amino acid precursors such as isoamyl alcohol and 2‐phenylethanol (C. Wang et al. 2020a), collectively indicating that microbial complementarity, rather than simple population dominance, is a key determinant of fermentation performance and flavor complexity in coffee systems.

In a wet‐processing study, Arabica cherries were fermented in 200 L HDPE bombonas under anaerobic conditions at temperatures below 21°C for up to 94 h (Dorta et al. 2024). When inoculated as a monoculture, *P*. *kluyveri* populations increased approximately 2.4‐fold, pH decreased from ∼6.5 to 4.0–4.5, and Brix consumption reached 53% by 94 h. Monoculture fermentation also significantly reduced enterobacteria and molds while supporting moderate growth of indigenous LAB, indicating that *P*. *kluyveri* alone can effectively drive fermentation, acidification, and microbial succession. Sensory scores of 79.0—characterized by caramel and chocolate notes—confirmed flavor enhancement without co‐inoculants. Co‐inoculation with LAB maintained strong *P*. *kluyveri* growth but modulated the fermentation environment to promote more complex aroma development, emphasizing red fruit and floral notes and achieving a higher sensory score of 81.75 compared to LAB alone (81.0) or the spontaneous control (∼78.0). Careful temperature management (< 21°C) ensured consistent microbial activity, allowing *P*. *kluyveri* to dominate early fermentation stages and produce desirable metabolites, including organic acids, higher alcohols, and esters, which contributed to improved cup quality (Dorta et al. 2024). Similarly, a *P*. *kluyveri* strain has demonstrated significant biotechnological potential during a SIAF for both depulped and whole cherries (Batista et al. 2025, 2026). In the depulped beans, the inoculated coffees showed robust yeast survival, with *P*. *kluyveri* maintaining viable populations throughout the process. Volatile analysis revealed that fermentations with *P*. *kluyveri* were dominated by aldehydes, particularly furan‐2‐carbaldehyde, along with acids and alcohols. Sensory evaluation confirmed its impact, with *P*. *kluyveri*‐inoculated coffees scoring 84.66, higher than spontaneous fermentation (81.59). Notably, *P*. *kluyveri* imparted distinct fruity notes, such as red fruits and strawberry, that were absent in control fermentations (Batista et al. 2025). These results collectively confirm that *P*. *kluyveri* can actively modulate the chemical and sensory trajectory of coffee fermentation, functioning effectively as an independent starter culture, with co‐inoculation providing further aroma refinement. Nevertheless, the persistence of positive sensory outcomes across studies remains difficult to compare directly because fermentation conditions, inoculum strategies, and sensory protocols vary considerably between experiments. Notably, biomass increase alone is not always a reliable indicator of fermentation success; maintenance of yeast viability and metabolic activity is critical.

Beyond fermentation, *P*. *kluyveri* has demonstrated strong potential in post‐harvest biocontrol. Together with *Saccharomycetales* such as *M*. *guilliermondii*, it dominated the fungal community of green coffee beans during the first 6 months of storage in jute sacks (Broissin‐Vargas et al. 2018). Molecular analyses confirmed persistent suppression of filamentous fungal growth. However, after 6 months, *Wallemia* spp. became dominant, coinciding with increased OTA production, higher fungal infection, and bean discoloration, highlighting the limitations of endogenous *P*. *kluyveri* in sustaining long‐term biocontrol under standard warehouse conditions. This shift suggests that environmental conditions and storage ecology may ultimately override the competitive advantages of *P*. *kluyveri* during prolonged storage. The competitive dynamics suggest that *P*. *kluyveri* can suppress early fungal colonization through nutrient competition, hyphal attachment, extracellular enzyme production, and killing toxins, but environmental factors such as elevated temperature, high relative humidity, and accumulation of reducing sugars favor xerophilic filamentous fungi, resulting in bleaching, mycotoxin formation, and reduced green bean quality. Strategies such as strain selection, optimized inoculum size, or modified storage conditions are therefore required to sustain long‐term biocontrol activity and improve the safety and quality of stored coffee beans. Overall, while direct evidence consistently supports the aroma‐enhancing potential of *P*. *kluyveri* in coffee fermentation, studies remain descriptive and laboratory‐based, with limited mechanistic understanding of how microbial interactions, metabolic pathways, and environmental conditions collectively shape fermentation outcomes. Further standardized and multi‐omics‐driven studies are therefore needed to validate its functional role under industrial coffee processing conditions. Table 4 outlines the key functions of *P*. *kluyveri* in fermented foods and beverages and their implications for coffee fermentation processes.

**TABLE 4:** Roles of *Pichia kluyveri* in fermented foods and beverages, with implications for coffee fermentation.

Food/beverage	Functional roles/key contributions	Key metabolites/compounds	Sensory/quality impact	References	Lessons/implications/notes
Wine/beer	Produces moderate ethanol (∼3.2%–5% v/v); releases thiol‐derived esters; increases acetate esters (25%–60%); reduces higher alcohols (∼15%) to decrease harshness; sequential/mixed inoculations modulate aroma formation	Acetate esters (3‐SHA, 2‐phenylethyl acetate); higher alcohols (hexanol, 2‐phenylethanol); ethyl esters (ethyl lactate, isoamyl acetate)	Fruity, floral, passionfruit; clear expression of varietal character; balanced flavor with less harshness	Amaya‐Delgado et al. (2013); Anfang et al. (2009); Á. Benito et al. (2019); Dutraive et al. (2019); K. Hu, Zhao, Edwards, et al. (2022); K. Hu, Zhao, Kang, et al. (2022); Huang et al. (2024); Rong et al. (2025); Ruiz et al. (2019); SaerensJan and Swiegers (2014); Waterhouse et al. (2024)	Moderate ethanol production and ester formation can be leveraged in coffee to enhance fruity and floral notes without excessive alcoholic, acidification or off‐flavors. Sequential or co‐inoculation strategies are critical for maximizing aroma impact; resilience in SmF
Coffee (wet)	Adapts well to wet coffee fermentation, contributing to mucilage degradation via pectinolytic activity, controlled acidification, and aroma compound production. Exhibits VOC‐mediated suppression of spoilage microbes and can function as a primary starter or auxiliary fermenter. Co‐inoculation with *Saccharomyces cerevisiae* or LAB enhances sugar utilization, microbial synergy, and volatile formation	Esters: isoamyl acetate (+43%–60%), 2‐phenylethyl acetate (up to 2.4‐fold increase). Alcohols: ethanol, isoamyl alcohol, 2‐phenylethanol. Organic acids: malic, acetic, oxalic. Aldehydes: furan‐2‐carbaldehyde	Fruity and floral aromas (red fruit, strawberry), along with chocolate, caramel, honey, nutty, and brown sugar notes. Enhanced flavor complexity and balanced acidity. Cupping scores typically 78–82+, with improvements compared with spontaneous fermentations	Batista et al. (2025); Dorta et al. (2024); Mahingsapun et al. (2022); C. Wang et al. (2020a, 2020b)	*P*. *kluyveri* is a versatile starter for coffee, enhancing ester‐mediated fruity/floral notes, controlling acidity, suppressing spoilage fungi, and improving sensory profiles. Inoculum size, altitude, temperature, and co‐inoculation strategies are key to reproducibility and aroma complexity
Coffee post‐harvest storage	Dominates early microbial colonization of green beans; limits growth of filamentous fungi and mycotoxin production; persists in short‐term storage but declines over 6 months	Biocontrol via nutrient competition, enzyme activity, killer toxins)	Maintains bean quality, reduces off‐flavors and mold contamination	Broissin‐Vargas et al. (2018)	Short‐term biocontrol potential; indicates need for optimized inoculum, strain selection, or modified storage conditions to maintain long‐term bean safety and quality

### 3.4 P. fermentans

#### 3.4.1 Fermented Beverages

*P*. *fermentans*, originally isolated from soil, has been widely investigated for its metabolic versatility and sensory contributions in alcoholic fermentation. Inoculation studies in pasteurized blueberry must (10^6^ CFU/mL, 28°C, 80 rpm) over 16 days revealed efficient sugar and acid metabolism: total sugars decreased from 12.25 ± 0.35 g/100 g to 1.83 ± 0.07 g/100 g, and ethanol reached 6.25 ± 1.15% (v/v). Deacidification was pronounced, with citric, malic, and tartaric acids reduced by 43.35%, 35.29%, and 44.68%, respectively. These findings are supported by earlier mechanistic studies demonstrating that *P*. *fermentans* efficiently metabolizes both glucose and citric acid under controlled shaking and static conditions. Most sugars were depleted within 24 h, while citric acid degradation was strongly affected by oxygen availability: under shaking, citric acid was almost completely consumed within 120 h, whereas static cultures showed slower degradation, with incomplete equilibrium even after 216 h. These findings demonstrate that *P*. *fermentans* can simultaneously utilize glucose and citric acid, highlighting its potential to reduce citric acid levels in fruit‐based fermentations such as blueberry wine. Moreover, enhanced aeration further accelerated citric acid metabolism, suggesting that fermentation conditions could be optimized to maximize this deacidification activity (Zhong et al. 2020, 2021). Sensory and electronic nose analyses further confirmed that wines fermented with *P*. *fermentans* exhibited greater sweetness, enhanced aroma intensity, and overall harmony, along with lower perceived acidity, bitterness, astringency, and hotness, compared with *S. cerevisiae* fermentations (Zhong et al. 2021). Similar findings have been reported in grape must, orange juice, and kiwifruit fermentations, where *P*. *fermentans* promoted a favorable balance between higher alcohols and ethyl acetate, yielding low‐alcohol beverages with enhanced aromatic and sensory properties (Kong et al. 2019; Mingorance‐Cazorla et al. 2003; Zhong et al. 2020). In most cases, sensory evaluations consistently ranked *P*. *fermentans*‐fermented products highest among multiple yeast strains, underscoring its capacity to enhance aroma balance and consumer acceptability.

Compared with *S. cerevisiae*, *P*. *fermentans* displays distinctive yet complementary fermentation traits. While *S. cerevisiae* dominates industrial wine production due to its high ethanol yield (> 10%), *P*. *fermentans* typically produces ∼5% ethanol and tolerates up to ∼6%, indicating suitability for early fermentation stages or mixed‐culture systems (Clemente‐Jimenez et al. 2005). When inoculated at 10^6^ CFU/mL, *P*. *fermentans* generated elevated levels of glycerol, 2,3‐butanediol, and characteristic aroma compounds such as 1‐hexanol, all contributing to improved mouthfeel and varietal expression. Sequential fermentations, in which *P*. *fermentans* preceded *S. cerevisiae* by 2–8 days, revealed complementary metabolic interactions: acetoin produced by *P*. *fermentans* was further metabolized by *S. cerevisiae*, while elevated glycerol and higher alcohol levels enhanced flavor complexity. Notably, *P*. *fermentans* produced significantly less acetic acid, thereby lowering volatile acidity and improving sensory balance (Clemente‐Jimenez et al. 2005).

The ecological adaptability of *P*. *fermentans* and its interaction with LAB are further illustrated by studies on Boza. LAB populations typically reached 8.62 log CFU/g, while yeasts averaged 4.48 log CFU/g, with *P*. *fermentans* representing 27.3%–59.9% of isolates (Mingorance‐Cazorla et al. 2003). Strains of *P*. *fermentans* efficiently deaminated l‐phenylalanine, producing high levels of 2‐phenylethanol that imparted a characteristic rose‐like aroma. Their carbohydrate utilization patterns, preferentially assimilating glucose and xylose but not maltose or sucrose, reduced competition for carbon sources with heterofermentative LAB, enabling a stable mixed microbial population. Unlike sucrose‐fermenting *S. cerevisiae*, which is inhibited by LAB‐derived acetic acid at typical Boza pH, *P*. *fermentans* coexisted harmoniously with LAB, supporting balanced growth and metabolite production that preserved Boza's traditional flavor and texture. Thus, *P*. *fermentans* strains with low invertase activity dominate Boza fermentation, generating high levels of 2‐phenylethanol while producing minimal ethanol, making them attractive for industrial applications seeking to standardize fermentation without compromising sensory authenticity (Mingorance‐Cazorla et al. 2003). Further mixed fermentation studies have revealed that *P*. *fermentans* supports LAB stability and performance. In co‐fermentations with *L. brevis*, *P*. *fermentans* populations peaked at ∼7.5 log CFU/mL on Day 1 and declined by Day 8, whereas *L*. *brevis* maintained higher viability (exceeding 8.0 log CFU/mL within 0–5 days) than in yeast‐free fermentations, where ethanol accumulation caused a rapid decline (K. Hu, Zhao, Kang, et al. 2022). This stabilization effect was attributed to yeast‐derived mannoproteins or cell wall polysaccharides that mitigated ethanol stress and prolonged LAB viability. Synergistic benefits are also evident in wine fermentations involving *P*. *fermentans*, *S. cerevisiae*, and *Oenococcus oeni*. in Merlot wine vinification, co‐fermentation of *P*. *fermentans* with *S. cerevisiae* and *O. oeni* enhanced ester formation, improved fruity aroma profiles, and promoted simultaneous alcoholic and malolactic fermentation, reducing overall fermentation time by approximately 45% (Q. Wang et al. 2018). These outcomes were linked to lower ethanol stress and improved compatibility between *P*. *fermentans* and *O*. *oeni*, which increased bacterial growth 1.7‐fold compared with control fermentations. Volatile analysis showed elevated concentrations of esters such as 3‐methylbutyl acetate, ethyl 3‐methylbutanoate, ethyl hexanoate, and ethyl octanoate, all compounds associated with enhanced fruity and floral sensory attributes (Zhao et al. 2022).

The influence of the *P*. *fermentans* inoculation ratio on aroma formation was clearly demonstrated in mixed fermentations with *S. cerevisiae* (Ma et al. 2017). Treatments with inoculum ratios of 1:10, 1:4, 1:1, 4:1, and 10:1 (where “1” corresponds to 10^6^ CFU/mL) yielded significantly higher total aroma compound levels compared with *S. cerevisiae* alone. Increasing *P*. *fermentans* inoculation enhanced the formation of acetate esters, medium‐chain fatty acids, and their corresponding ethyl esters, whereas lower ratios favored phenylethyl and short‐chain fatty acid ethyl esters. Sensory analysis revealed that mixed fermentations produced wines with richer, more complex aroma profiles. Sweet and acid‐fruity notes exceeded 40% intensity in high‐ratio treatments, while floral aromas peaked (45%) in the 1:4 ratio, reflecting optimal formation of phenylethyl and short‐chain ester compounds. At the highest ratio (10:1), however, an undesirable earthy note (∼22% intensity) emerged, likely from fatty acid accumulation. These findings emphasize the importance of optimizing *P*. *fermentans* inoculation levels to enhance desirable fruity and floral characteristics while minimizing off‐flavor formation. These beverage‐derived findings provide a mechanistic context for interpreting *P*. fermentans' potential contributions to coffee fermentation, particularly regarding deacidification, ester production, and LAB compatibility.

#### 3.4.2 Extending Lessons for *P. fermentans* to Coffee

*P*. *fermentans* is a metabolically versatile non‐*Saccharomyces* yeast that efficiently metabolizes glucose and citric acid and functions as an effective biocatalyst for flavor ester production under controlled conditions (Table 5). Extrapolating from beverage systems, its performance is strongly influenced by inoculation dose and strain ratios in mixed fermentations, and it enhances flavor complexity without exerting excessive fermentative dominance, enabling complementary function alongside *S. cerevisiae* and LAB. In this respect, *P*. *fermentans* occupies a functionally distinct niche relative to other *Pichia* species: unlike *P*. *kudriavzevii*, which establishes dominance through exceptional stress tolerance and survives well into advanced fermentation stages, or *W*. *anomalus*, which exerts strong biocontrol through killer toxin production, *P*. *fermentans* derives its competitive advantage primarily from early‐stage metabolic activity, deacidification capacity, and synergistic compatibility with LAB under moderately aerobic conditions (Clemente‐Jimenez et al. 2005; Q. Wang et al. 2018; Zhong et al. 2020). This distinction has direct practical implications for coffee fermentation design, although whether these functional roles translate equivalently to coffee matrices remains to be directly verified.

**TABLE 5:** Functional roles of *Pichia fermentans* across beverage, food, and coffee fermentations.

Food/beverage system	Functional roles/key contributions	Key metabolites/compounds	Sensory/quality impact	References	Lessons/implications/notes
Fruit wines and low‐alcohol beverages (blueberry, grape, citrus, kiwi)	Efficient glucose and citric acid utilization; strong deacidification capacity (citric, malic, tartaric acids); moderate ethanol production (∼5%–6%); lower acetic acid than *Saccharomyces cerevisiae*	Ethanol; glycerol; 2,3‐butanediol; ethyl acetate; higher alcohols (1‐hexanol, 2‐phenylethanol)	Reduced acidity and astringency; improved sweetness and mouthfeel; enhanced aroma harmony and consumer acceptability	Clemente‐Jimenez et al. (2005); Kong et al. (2019); Mingorance‐Cazorla et al. (2003); Zhong et al. (2020, 2021)	Demonstrates strong potential for controlled acid modulation; *P*. *fermentans* functions primarily as a cooperative, modulatory yeast rather than a dominant fermenter
Mixed wine fermentations with *S. cerevisiae*	Complements *S. cerevisiae* in sequential inoculation; produces glycerol and acetoin metabolized later by *S. cerevisiae*; low volatile acidity; supports balanced alcoholic fermentation	Acetoin; glycerol; esters (ethyl acetate, ethyl hexanoate); higher alcohols	Increased flavor complexity; reduced volatile acidity; enhanced varietal expression	Clemente‐Jimenez et al. (2005); Ma et al. (2017)	*P*. *fermentans* consistently occupies early to intermediate fermentation niches, where its moderate fermentative capacity, broad substrate utilization, and strong metabolic plasticity enable it to shape fermentation trajectories without overwhelming microbial consortia
Cereal‐based beverages (Boza)	Coexists stably with LAB; deaminates l‐phenylalanine; produces minimal ethanol; avoids competition for sucrose/maltose	2‐Phenylethanol	Rose‐like floral aroma; preservation of traditional sensory profile	Mingorance‐Cazorla et al. (2003)	Highlights compatibility with LAB and suitability for low‐ethanol, aroma‐driven fermentations, relevant for coffee where ethanol should remain limited
LAB‐yeast mixed fermentations (wine, cereal systems)	Stabilizes LAB populations; produces cell‐wall polysaccharides (mannoproteins); reduces ethanol stress on LAB; accelerates malolactic fermentation	Esters (3‐methylbutyl acetate, ethyl hexanoate); polysaccharides	Enhanced fruity/floral aroma; shorter fermentation time	K. Hu, Zhao, Kang, et al. (2022); Q. Wang et al. (2018); Zhao et al. (2022)	Indicates microbial buffering capacity, useful for stabilizing LAB‐yeast interactions in coffee fermentations
Coffee—Laboratory wet fermentation	Strong growth (up to 7.7 log CFU/Ml, monoculture); produces yeast‐derived volatiles; limits bacterial overgrowth; reduces excessive acidification	Ethanol; ethyl acetate; isoamyl acetate; 2,3‐butanedione	Strong fruity and buttery aromas; enhanced fermented notes	de Melo Pereira et al. (2014)	Confirms direct aroma‐enhancing role in coffee without overacidification, and the essential of process control (inoculum size and ratio, strain compatibility, air)
Coffee—Pilot and field scale wet fermentation	Dominates indigenous microbiota; produces restricted microbial community; suppresses LAB proliferation; enhances yeast‐specific volatiles	Ethanol; acetaldehyde; isoamyl acetate; ethyl acetate	Floral, vanilla, fruity notes; improved cup clarity	de Melo Pereira et al. (2015)	Demonstrates suitability as a process‐control starter for reproducible coffee fermentation
Coffee—Monoculture and LAB co‐inoculation	Monoculture: volatile enrichment, controlled pH; LAB co‐inoculation: synergistic aroma complexity; sequential LAB → yeast most effective	Phenylethyl alcohol; benzyl alcohol; ethyl linoleate	High aroma complexity; floral and fruity dominance	de Mello Sampaio et al. (2025)	Identifies inoculation strategy as a key for high‐value coffee

Abbreviations: AAB, acetic acid bacteria; CFU, colony‐forming units; LAB, lactic acid bacteria; VOC, volatile organic compounds.

Direct evidence from coffee confirms that laboratory‐scale wet fermentations inoculated with *P*. *fermentans* at 6 log CFU/mL over 48 h at 28°C resulted in growth to 7.74 log CFU/mL and production of ethanol, ethyl acetate, isoamyl acetate, 2,3‐butanedione, and hexanal (de Melo Pereira et al. 2014). Specifically, *P*. *fermentans* produced ethanol (≈1–43 mg/g), ethyl acetate (≈ND–42 mg/g), isoamyl acetate (≈ND–10 mg/g), 2,3‐butanedione (≈8–18 mg/g), and hexanal (≈13–22 mg/g). Relatively open headspace in this experimental setup likely facilitated oxygen availability, supporting both growth and ester formation. Sensory evaluation revealed that coffee fermented with *P*. *fermentans* exhibited the highest intensity of fruity aroma compared with spontaneous controls (≈6–8/10), while buttery and fermented notes were also significantly enhanced (≈5–6/10). When co‐inoculated with *Saccharomyces* sp. at a 1:1 ratio, *P*. *fermentans* growth was reduced to 3.26 log CFU/mL after 48 h, while the *Saccharomyces* sp. reached over 7 log CFU/mL, suggesting competitive or antagonistic interactions and highlighting the importance of inoculation strategy and strain compatibility. Despite this suppression, mixed fermentations still produced substantial volatile compounds, with ethanol reaching ≈89 mg/g, while ethyl acetate and isoamyl acetate levels decreased. Sensory evaluation indicated a slight reduction in fruity aroma (score ≈ 6), whereas buttery and fermented notes remained largely unchanged (de Melo Pereira et al. 2014). This competitive suppression of *P*. *fermentans* by *Saccharomyces* sp. in coffee fermentation directly contradicts the broadly synergistic coculture dynamics reported for the same species combination in wine and Merlot fermentations (Clemente‐Jimenez et al. 2005; Q. Wang et al. 2018), where *P*. *fermentans* consistently enhanced ester formation and sensory complexity without being outcompeted. This inconsistency most plausibly reflects differences in substrate sugar composition, inoculation ratio, and the more nutrient‐limited nature of coffee fermentation liquid relative to grape must, rather than a fundamental biological incompatibility. Critically, this finding cautions against direct extrapolation of coculture strategies optimized in beverage fermentation systems to coffee without substrate‐specific validation and underscores the need for systematic inoculation ratio studies in coffee‐specific matrices.

**FIGURE 4:**
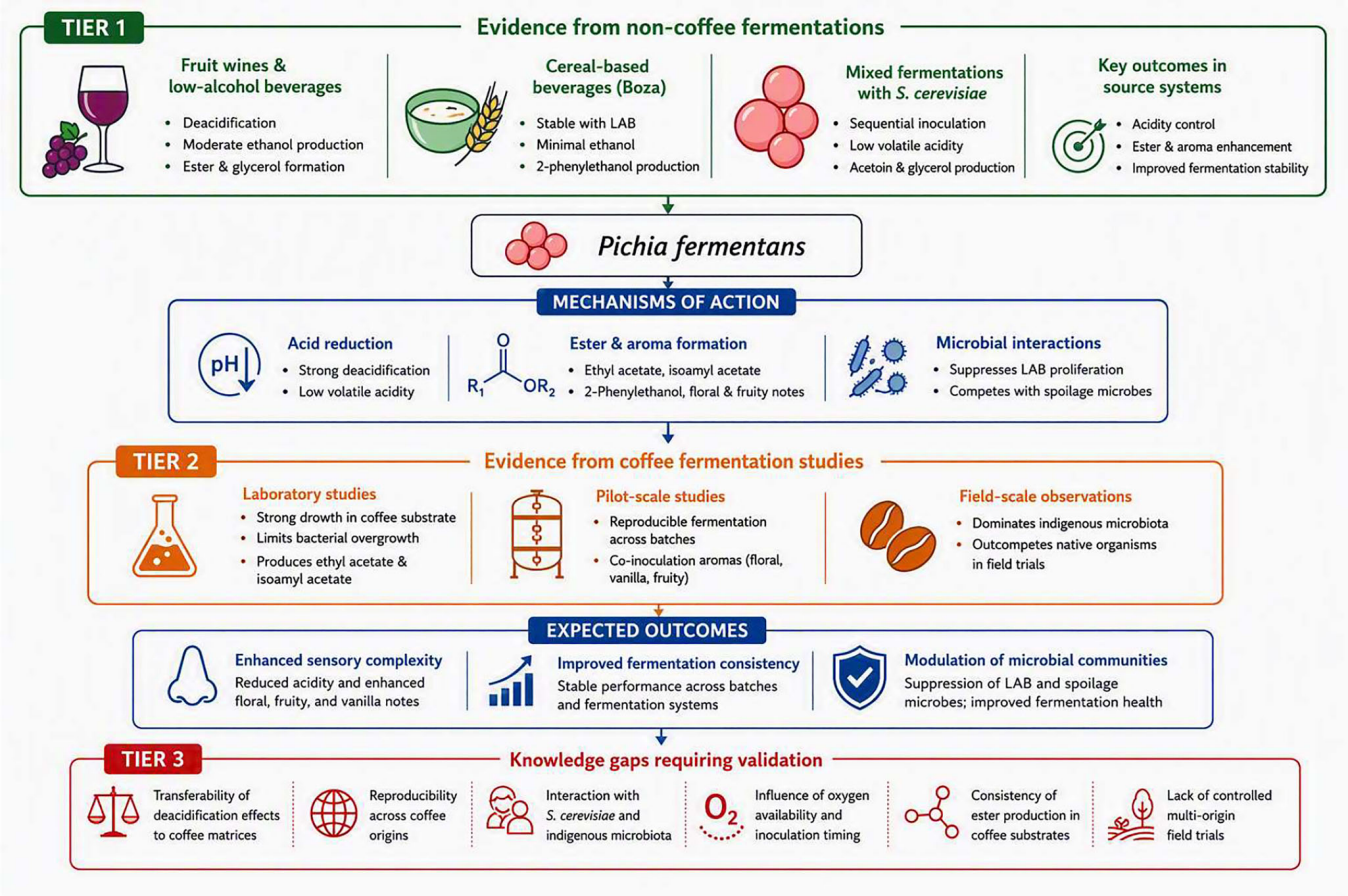
Functional roles of *Pichia fermentans* across fermentation systems.

Encouraged by these laboratory‐scale results, *P*. *fermentans* was implemented as a starter culture in field‐scale wet coffee fermentations (de Melo Pereira et al. 2015). Inoculation successfully dominated the indigenous microbiota, leading to a more restricted microbial composition and increasing yeast populations from 7.5 to 8.8 log CFU/mL. This dominance enhanced the production of yeast‐derived volatiles, including ethanol, acetaldehyde, ethyl acetate, and isoamyl acetate, while reducing lactic acid formation. Sucrose supplementation did not significantly affect *P*. *fermentans* growth or persistence but maintained higher levels of wild bacteria and lactic acid, similar to spontaneous fermentations. Initial pH values (∼5.4) decreased differently depending on treatment: spontaneous fermentations reached 4.0, correlating with higher bacterial populations (6.22 log CFU/mL), whereas inoculated fermentations ended at pH 4.4 with lower bacterial loads (5.68 log CFU/mL). This indicates that *P*. *fermentans* inoculation limits bacterial proliferation, moderating acidification. Roasted beans showed no significant differences in sugars or organic acids across treatments, suggesting that yeast metabolism primarily influenced the volatile fraction rather than the bulk composition. Notably, inoculated fermentations enriched roasted beans with yeast‐specific aroma compounds, translating into distinctive sensory attributes, including pronounced floral notes and intense vanilla flavor (de Melo Pereira et al. 2015). Recent studies further emphasize the dual potential of *P*. *fermentans* as both a monoculture inoculant and a component of mixed‐culture strategies in coffee fermentation. Monoculture inoculation effectively outcompeted indigenous yeasts, producing a restricted microbial community, lower bacterial loads (∼5.7 log CFU/mL), and higher final pH (∼4.4) compared with spontaneous fermentations (∼6.2 log CFU/mL, pH 4.0), indicating suppression of lactic acid‐driven acidification (de Mello Sampaio et al. 2025). These fermentations also enhanced yeast‐derived volatiles such as ethanol, acetaldehyde, ethyl acetate, and isoamyl acetate while decreasing lactic acid accumulation, improving the balance of metabolic outputs. Greater benefits were observed when *P*. *fermentans* was co‐inoculated with LAB. In combination with *Pediococcus pentosaceus*, both wild yeasts and bacteria were suppressed, stabilizing the fermentation process. Sequential inoculation*—*LAB for 36 h followed by *P*. *fermentans—*was particularly effective, generating approximately threefold higher volatile content than spontaneous fermentation. Gas chromatography detected 93 volatile compounds, including ethyl linoleate, 1‐hexanol, 2‐heptanol, benzeneacetaldehyde, benzyl alcohol, and phenylethyl alcohol. Sensory evaluations confirmed that co‐inoculated treatments produced beverages with enhanced aroma complexity, characterized by floral and fruity notes, and achieved higher overall quality scores compared with monoculture or spontaneous fermentations (de Mello Sampaio et al. 2025). However, it is worth noting that de Melo Pereira et al. (2014) reported reduced fruity aroma intensity in co‐inoculated treatments relative to *P*. *fermentans* monoculture, while de Mello Sampaio et al. (2025) reported the opposite—greater aroma complexity under co‐inoculation. These contradictory outcomes likely reflect differences in the identity and inoculation timing of the co‐inoculated partner, fermentation scale, and oxygen availability across the two studies and collectively highlight that the benefits of co‐inoculation with *P*. *fermentans* in coffee are highly context‐dependent and cannot be assumed universally positive without careful experimental design. Despite the promising evidence reviewed here, the current understanding of *P*. *fermentans* in coffee fermentation remains constrained by several limitations. Coffee‐specific studies are limited in number and scale, with only one field‐scale implementation reported (de Melo Pereira et al. 2015), and significant strain‐level variability in ester production, stress tolerance, and competitive fitness has yet to be systematically addressed in coffee‐specific matrices. Key process variables, including oxygen availability, inoculation timing, and indigenous microbiota composition, remain poorly characterized in this context, and no studies have evaluated *P*. *fermentans* under modern processing methods, such as anaerobic fermentation or carbonic maceration. Controlled, multivariable studies across diverse coffee origins and processing conditions will be essential before this species can be reliably deployed as a defined starter culture in commercial coffee bioprocessing. Figure 4 summarizes the cross‐system evidence supporting its applications.

### 3.5 M. guilliermondii

#### 3.5.1 Fermented Beverages

*M*. *guilliermondii* is frequently detected at high abundance in natural grape must fermentations, reflecting its important ecological role in shaping wine aroma profiles. Laboratory‐scale studies have demonstrated the functional potential of *M*. *guilliermondii* strains. In sequential inoculation with *S. cerevisiae*, *M*. *guilliermondii* can achieve high cell densities during the early stages of fermentation and exhibits strong β‐glucosidase activity, releasing bound aroma precursors such as terpenes and C13‐norisoprenoids. This glycosidase activity operates through hydrolysis of glycosidic bonds in bound aroma precursors, with the enzyme repertoire encompassing β‐glucosidase, β‐xylosidase, and α‐l‐rhamnosidase, collectively liberating volatile aroma‐active compounds, including monoterpenols such as geraniol and linalool, as well as 2‐phenylethanol from their glycosylated precursor forms (Rodríguez et al. 2004, 2007, 2010). This enzymatic activity leads to elevated concentrations of esters, higher alcohols, and fatty acids, supporting its classification as a “flavor yeast” (Gao et al. 2022). Subsequent pilot‐scale fermentation trials using Chardonnay must confirm these observations (Mi et al. 2025). Inoculated before *S. cerevisiae*, *M*. *guilliermondii* rapidly colonized the must in the early fermentation stages, followed by a decline in population due to ethanol accumulation and microbial competition, without inhibiting *S. cerevisiae* performance. Sequential inoculation enhanced key aroma compounds, including ethyl acetate, acetate esters, and 2‐phenylethanol, contributing rose, honey, and fruity notes. Characteristic esters such as ethyl caprylate and ethyl caprate were also produced, while volatile phenols remained below sensory thresholds. Similar functional effects have been observed in other wine varieties. In Cabernet Sauvignon fermentations, sequential inoculation of *M*. *guilliermondii* before *S. cerevisiae* produced a characteristic rise‐fall pattern in yeast populations (Zang et al. 2023). *M*. *guilliermondii* reached a maximum biomass of 8 × 10^7^ CFU/mL at 48 h before declining to undetectable levels by the end of fermentation, while *S. cerevisiae* subsequently dominated with populations of approximately 10^5^ CFU/mL. Wines produced under this regime exhibited slightly reduced ethanol (13.95%–14.44% v/v) and glycerol concentrations compared with controls, while total and volatile acidity remained within acceptable oenological ranges. HS–SPME–GC‒MS analysis identified 64 volatile compounds, including 18 alcohols, 31 esters, 7 terpenes, 2 acids, and 6 aldehydes/ketones/others. Notably, higher alcohol concentrations, dominated by isoamyl alcohol and 2‐phenylethanol, remained below 300 mg/L, contributing banana, honey, rose, and clove aromas. Ester formation was markedly enhanced, including increases in isoamyl acetate (+123%), ethyl heptanoate (+621%), ethyl octanoate (+37%), *trans*‐4‐decenoate (+238%), and ethyl propionate (+140%), while isoamyl butyrate and isoamyl octanoate were detected exclusively in *M*. *guilliermondii*‐inoculated wines. Terpene concentrations, including damascone, increased by 171.43% during aging, while fatty acids were limited to hexanoic acid, reducing harsh sensory attributes (Zang et al. 2023).

Beyond ester and terpene enhancement, *M*. *guilliermondii* has demonstrated intrinsic capacity for monoterpene synthesis. A multi‐stress‐tolerant strain produced nerol, an acyclic monoterpene with floral and citrus notes, at approximately 2.74 mg/L, levels exceeding those achieved in engineered *Escherichia coli* systems (Mo et al. 2021). Nerol biosynthesis proceeds via geranyl diphosphate intermediates derived from central carbon metabolism, with enzymatic contributions from geranyldiphosphatase, monoterpenyl diphosphatase, and geraniol isomerase, representing a de novo terpene biosynthesis capacity that is relatively rare among non‐*Saccharomyces* yeasts. Treated wines showed elevated isoamyl acetate, ethyl heptanoate, ethyl octanoate, *trans*‐4‐decenoate, linalool, nerol, and damascene, confirming the yeast's ability to enhance aromatic diversity during early fermentation stages (Mo et al. 2021). Overall, *M*. *guilliermondii* has been reported to contribute to early‐stage fermentation by enhancing the aroma and flavor of fermented beverages through esters and terpenes and releasing bound aroma precursors while coexisting with *S. cerevisiae* without impairing fermentation.

In micro‐fermentations conducted in blonde beer wort (17°Bx, pH 4.5, supplemented with Zn^2+^ and hop extract), *M*. *guilliermondii* exhibited limited fermentative activity, consistent with its slow sugar consumption and minimal ethanol production relative to *Saccharomyces* strains (Sampaolesi et al. 2023). Fermentations were performed at 20°C in 70 mL wort with an inoculum of 2 × 10^6^ cells/mL under mild aeration, simulating realistic brewing conditions. Although the strain did not fully metabolize wort sugars, it demonstrated robust growth in high‐sugar environments, assimilating maltose and maltotriose and producing 2,3‐butanediol. Its metabolic activity favored aroma compound synthesis, including the conversion of 2‐phenylethanol into its ester (rose/honey notes) and the formation of ethyl esters up to 0.44 ± 0.06 mg/L, alongside substantial production of ethyl hexanoate, octanoic, and decanoic acids, contributing fruity and floral aromas. In mixed fermentations with *S. cerevisiae* (sequential or simultaneous inoculation at a 1:9 ratio), *S. cerevisiae* efficiently completed sugar fermentation and increased ethanol yield, while *M*. *guilliermondii* enhanced the ester profile. Notably, ethyl acetate and short‐chain fatty acid esters (apple, berry, floral notes) were significantly elevated (*p* < 0.05), and ethyl decanoate concentrations exceeded their sensory thresholds, producing a beer with a more pronounced fruity and floral character (Sampaolesi et al. 2023). These beverage‐derived findings provide a mechanistic context for interpreting *M*. *guilliermondii*'s potential aroma‐enhancing contributions in coffee fermentation, particularly its glycosidase activity and early‐stage volatile production.

#### 3.5.2 Extending Lessons for *M. guilliermondi* to Coffee

Extrapolating from wine and beer (Table 6), *M*. *guilliermondii* is distinctive in its combination of strong glycosidase activity, de novo monoterpene biosynthesis capacity, and notable stress tolerance—including ethanol tolerance up to 20% and strong osmotic resistance—traits that collectively position it as a flavor‐liberating and early‐acting yeast rather than a dominant fermentation driver (Khattab and Kodaki 2016; Sampaolesi et al. 2023). This functional profile is extrapolated from beverage systems; whether it translates equivalently to coffee matrices remains to be directly verified. This profile contrasts with *P*. *kudriavzevii*, which dominates through stress resilience and active ester synthesis, and with *W*. *anomalus*, which exerts stronger process control through killer toxin‐mediated biocontrol, suggesting that *M*. *guilliermondii* is best deployed as a precision aroma tool rather than a primary inoculant.

**TABLE 6:** Fermentation roles, functional traits, and cross‐domain lessons of *Meyerozyma guilliermondii* with implications for coffee fermentation.

Fermentation system	Role/function of *M*. *guilliermondii*	Key metabolic or technological traits	Observed outcomes	Key references	Lessons/implications/notes
Wine (Cabernet Sauvignon)	Early‐stage non‐*Saccharomyces* modulator	Rapid early growth (up to 8 × 10^7^ CFU/mL), sequential decline; ester and terpene production; low ethanol yield	Reduced ethanol; increased esters (isoamyl acetate, ethyl heptanoate, ethyl octanoate); enhanced floral, fruity, spicy aromas; controlled fatty acids	Gao et al. (2022); Mi et al. (2025); Zang et al. (2023)	Suggests suitability for early inoculation in coffee to shape aroma precursors without dominating fermentation or altering acidity under SmF
Wine/terpene biosynthesis systems	Aroma enhancer via terpene synthesis	Nerol biosynthesis from central carbon metabolism; stress tolerance	Production of nerol (∼2.74 mg/L); increased linalool, damascone; enhanced aromatic diversity	Gao et al. (2022); Mi et al. (2025); Mo et al. (2021)	Indicates potential to enhance floral‐citrus aroma notes in coffee under controlled fermentation
Beer (blonde wort)	Aroma‐enhancing co‐fermentation yeast	Growth in high‐sugar environments; maltose/maltotriose assimilation; low ethanol production; 2,3‐butanediol synthesis; ester formation including 2‐phenylethyl acetate, ethyl hexanoate, octanoate, decanoate	Minimal ethanol on its own; enhanced fruity and floral aroma in co‐fermentation with *Saccharomyces cerevisiae*; ethyl decanoate above sensory threshold; increased short‐chain fatty acid esters	Sampaolesi et al. (2023)	Acts primarily to enrich beer flavor complexity; suitable for co‐inoculation strategies to improve aroma without compromising fermentation efficiency
Wet coffee fermentation (depulped beans)	Aroma fine tuner	Moderate yeast growth; selective volatile modulation	Detection of 2‐heptanone, 2‐nonanol, methyl salicylate; increased roasted, caramel, spicy notes after roasting; minimal sensory score change	Gonçalves Bravim et al. (2023)	Indicates role as aroma modifier rather than kinetic driver in wet coffee fermentation
Self‐induced anaerobiosis fermentation (whole cherries)	Aroma complexity enhancer	Ester and phenolic compound modulation under anaerobic conditions	Increased ester and aldehyde fractions; improved sensory scores, especially in *Coffea canephora*	do Rosário et al. (2024)	Demonstrates higher impact under controlled or anaerobic systems, suggesting process‐specific optimization strategies

Abbreviations: CFU, colony‐forming units; HS–SPME–GC–MS, headspace solid‐phase microextraction–gas chromatography–mass spectrometry; SIAF, self‐induced anaerobic fermentation; VOC, volatile organic compounds.

Direct evidence from coffee confirms that during wet fermentation of depulped beans, *M*. *guilliermondii* inoculation led to slightly lower pH values (4.12 after 48 h) and the exclusive detection of lactic acid in inoculated treatments, while yeast populations increased by approximately one log cycle during the first 24 h and remained stable thereafter (Gonçalves Bravim et al. 2023). Yeast populations increased by approximately one log cycle during the first 24 h and remained stable thereafter, with no significant impact on filamentous fungal populations. Volatile analysis of green beans revealed yeast‐specific metabolites, including 2‐heptanone and 2‐nonanol, while methyl salicylate (contributing minty, sweet, or wintergreen‐like notes) persisted only in *M*. *guilliermondii*‐treated samples. In roasted coffee, inoculated treatments showed higher relative abundances of *p*‐ethylguaiacol, furfuryl alcohol, furfural, 5‐methylfurfural, and maltol, and the exclusive detection of 2‐ethyl‐5‐methylpyrazine, contributing to roasted, nutty, caramel, and spicy attributes. The elevation of *p*‐ethylguaiacol in roasted inoculated beans is consistent with the cinnamate decarboxylase‐mediated phenolic metabolism documented in *M*. *guilliermondii*, whereby *p*‐coumaric acid is decarboxylated to 4‐vinylphenol intermediates that are subsequently transformed into roasted phenolic volatiles during roasting (Sangorrín et al. 2013). Sensory evaluation classified all coffees as “premium,” with *M*. *guilliermondii*‐treated coffee scoring 78.77 versus 78.73 for the control, suggesting aroma fine‐tuning rather than major sensory restructuring (Gonçalves Bravim et al. 2023). These findings indicate that *M*. *guilliermondii* exerts a moderate and targeted influence during wet coffee processing. Its contribution is primarily associated with the production or modulation of specific volatile compounds enhancing aroma complexity in both green and roasted beans, while exerting minimal impact on core physicochemical parameters or on overall sensory classification. The referenced study did not report the inoculum concentration or demonstrate starter culture dominance, as evidenced by the lack of a significant increase in total yeast populations encompassing both endogenous and inoculated communities. This limitation constrains the interpretation of the yeast's competitive performance during fermentation. Consequently, *M*. *guilliermondii* appears most suitable for fine‐scale aroma modulation within wet coffee fermentations, rather than as a dominant driver of fermentation kinetics or a determinant of major improvements in cup quality. Stronger effects were observed under self‐induced anaerobiosis fermentation (SIAF) of whole coffee cherries. In these controlled systems, yeast inoculation (6.6 log for 5 days) significantly modified volatile profiles, increasing ester content in *C. arabica* (21.66% vs. 20.53% in spontaneous fermentation) and elevating aldehydes (14.39% vs. 13.48%) and 4‐ethenyl‐2‐methoxyphenol (21.90% vs. 19.76%) in *Coffea canephora* (do Rosário et al. 2024). The marginal improvement under wet fermentation contrasts with significant sensory gains under SIAF of whole cherries, where *C*. *canephora* achieved specialty classification (do Rosário et al. 2024). This discrepancy likely reflects greater substrate accessibility for glycosidase and cinnamate decarboxylase activity under anaerobic mucilage‐rich conditions, underscoring that *M*. *guilliermondii*'s sensory contribution cannot be generalized across processing methods without method‐specific evaluation. Collectively, these findings demonstrate that *M*. *guilliermondii* functions as a highly adaptable, early‐acting yeast, contributing to aroma modulation and enzymatic enhancement of flavor precursors. The current understanding remains constrained by very few coffee‐specific studies, absent inoculation optimization data, the lack of strain characterization in coffee matrices, and no field‐scale evaluation. The generally absent pectinolytic activity in most strains (Deshpande 2019; Pando Bedriñana et al. 2012) further limits its utility as a standalone starter where mucilage degradation is needed, distinguishing it functionally from *W*. *anomalus* and *P*. *kluyveri*.

### 3.6 D. hansenii

#### 3.6.1 Fermented Beverages

*D*. *hansenii* has been successfully applied in both beer and wine fermentations, demonstrating its potential to influence sensory profiles. For example, in beer, *D*. *hansenii* was evaluated in a blond‐style ale using a 4 L wort prepared via the all‐grain brew in a bag (BIAB) method—a simplified craft brewing technique allowing all‐grain production without multiple vessels for mashing, lautering, and sparging (Ordulj et al. 2024). The wort was inoculated with *D*. *hansenii* and incubated at 20°C for 14 days, followed by a 3‐day cold crash at 4°C and 14 days of bottle aging. Sensory analysis revealed that beers fermented with *D*. *hansenii* exhibited distinctive malt and fruity aromas and contributed positively to mouthfeel attributes such as body and astringency. These sensory effects are likely linked to higher alcohol and ester biosynthesis through amino acid catabolism via the Ehrlich pathway, together with modulation of organic acid metabolism under oxygen‐limited brewing conditions (Liang et al. 2021; Valdez Castillo et al. 2021). Although the overall fermentation efficiency was lower than that of the *S. cerevisiae* control, *D*. *hansenii* demonstrated potential as a modulator of beer flavor suitable for co‐fermentation or specialty beer production.

In wine, *D*. *hansenii* was evaluated in sequential co‐fermentations with *S. cerevisiae* using pasteurized grape must (∼220 mL, inoculated at ∼1 × 10^7^ cells/mL), followed by *S. cerevisiae* addition at ∼1 × 10^6^ cells/mL after 3 days (van Wyk et al. 2020). While *D*. *hansenii* had minimal impact on general fermentation performance or ethanol production, it significantly influenced the non‐ester volatile fraction. Co‐fermentation enhanced the release of terpenes and norisoprenoids, which contribute floral, fruity, and varietal aromas, likely via glycosidase‐mediated liberation of aroma precursors from bound forms. β‐Glucosidase activity is considered a key mechanism underlying this effect, enabling the hydrolysis of glycosylated nonvolatile precursors into free aroma‐active monoterpenes and related compounds (Varela and Borneman 2017). Although acetate and ethyl esters were reduced, these results highlight *D*. *hansenii*'s capacity to modulate wine aroma beyond ester formation, enhancing varietal and sensory complexity (van Wyk et al. 2020). Across beer and wine, *D*. *hansenii* produced distinctive esters, terpenes, and norisoprenoids and modulated mouthfeel without necessarily affecting ethanol production. These functional properties appear to be closely associated with metabolic flexibility, including oxygen‐responsive carbon flux, amino acid‐derived volatile formation, precursor hydrolysis, and selective phenolic biotransformation pathways (Liang et al. 2021; Valdez Castillo et al. 2021). Its tolerance to diverse fermentation conditions, along with its ability to function in sequential or mixed‐culture systems, underscores its potential to support controlled, inoculated fermentations under submerged conditions, enhancing flavor consistency and broadening the spectrum of desirable sensory attributes. These beverage‐derived findings provide an extrapolated mechanistic context for considering *D*. *hansenii*'s potential role in coffee fermentation, particularly as an aroma‐modulating adjunct during later fermentation and drying stages.

#### 3.6.2 Expanding Knowledge for *D. hansenii* to Coffee

*D*. *hansenii* functions primarily as an aroma‐modulating rather than a primary fermentative yeast, consistently reshaping volatile profiles through enzymatic release of bound aroma precursors, amino acid catabolism, and selective formation of alcohols, esters, terpenes, and norisoprenoids. Its strong tolerance to osmotic, saline, and low water activity environments enables persistence under harsh conditions, supporting sustained aroma development during extended fermentations and drying (Table 7). Despite the frequent detection of *D*. *hansenii* in coffee fermentations across diverse geographical regions and processing methods (De Carvalho Neto et al. 2017; Elhalis et al. 2021c; Feng et al. 2016; Pérez‐Escalante et al. 2021), as shown earlier, no studies have yet reported its deliberate use as a starter culture to control or direct the coffee fermentation process, to the best of the authors' knowledge. Among the species reviewed here, *D*. *hansenii* occupies the most specialized functional niche: it is neither a prolific ester producer such as *P*. *kluyveri* and *W*. *anomalus* nor a stress‐resilient dominant fermenter such as *P*. *kudriavzevii* but rather excels as a precursor‐liberating, phenolic‐biotransforming, and aroma‐fine‐tuning adjunct yeast with exceptional tolerance to osmotic and low water activity stress (Valdez Castillo et al. 2021; van Wyk et al. 2020). This profile makes it uniquely suited to later fermentation stages or post‐fermentation environments such as drying, where most other *Pichia* species have declined and where sustained enzymatic activity on aroma precursors could meaningfully shape the final volatile profile of dried coffee beans.

**TABLE 7:** Fermentation roles, functional traits, and cross‐domain lessons of *Pichia hansenii* with implications for coffee fermentation.

Fermentation system	Role/function of *Debaryomyces hansenii*	Key metabolic or technological traits	Observed outcomes	Key references	Lessons/implications/notes
Beer (blond‐style ale)	Aroma and mouthfeel modulator	Low ethanol yield; flavor‐active metabolism; tolerance to brewing conditions	Enhanced malt and fruity aromas; increased body and astringency; lower fermentation efficiency than *Saccharomyces cerevisiae*	Ordulj et al. (2024)	Indicates suitability as a co‐fermentation or adjunct yeast to modulate flavor rather than drive coffee fermentation kinetics
Wine (grape must, sequential co‐fermentation)	Non‐ester aroma enhancer	Glycosidase activity; terpene and norisoprenoid liberation; limited ester production	Increased terpenes and norisoprenoids; reduced acetate and ethyl esters; enhanced varietal aroma expression	van Wyk et al. (2020)	Suggests potential to liberate bound aroma precursors in coffee mucilage and beans without excessive ester formation

Abbreviations: BIAB, brew in a bag; CFU, colony‐forming units; HS–SPME–GC–MS, headspace solid‐phase microextraction–gas chromatography–mass spectrometry; VOC, volatile organic compounds.

Its documented behavior across analogous fermentation systems allows several evidence‐based expectations to be proposed. First, *D*. *hansenii* is unlikely to act as a dominant ethanol‐producing organism in coffee fermentations. Instead, its role would most plausibly resemble that observed in wine and tea systems: a secondary or adjunct yeast that fine‐tunes aroma complexity rather than driving bulk fermentation. Coffee mucilage and pulp contain abundant glycosylated aroma precursors, amino acids, and organic acids, providing a suitable biochemical context for *D*. *hansenii*'s glycosidase, amino acid catabolic, and ester‐modulating activities. The absence of demonstrated dominance by *D*. *hansenii* in spontaneous coffee fermentations, despite its consistent detection, is consistent with its characteristically low ethanol yield and moderate competitive fitness under nutrient‐rich, actively fermenting conditions. Its persistence is more likely driven by osmotic and low water activity tolerance than fermentative competitiveness, suggesting that its ecological niche in coffee is primarily during drying and post‐fermentation stages rather than active mucilage fermentation (Valdez Castillo et al. 2021; van Wyk et al. 2020).

Second, *D*. *hansenii* could contribute to the liberation and transformation of bound volatiles, potentially increasing floral, fruity, and sweet‐associated compounds such as terpenes, higher alcohols, and phenyl‐derived aromatics while moderating excessive ester formation. This aligns with its demonstrated capacity to enhance non‐ester volatile fractions and promote matrix‐specific aroma expression in grape must and botanical fermentations. However, this expectation must be evaluated cautiously, as the substrate specificity of its glycosidases has been primarily characterized on grape‐derived precursors, and it remains unknown whether the glycosylated precursor profile of coffee mucilage would equally support this enzymatic activity. This represents a critical unresolved question that prevents direct extrapolation from beverage systems to coffee without targeted substrate‐specific investigation. Third, its stress tolerance, including resilience to osmotic pressure, fluctuating oxygen availability, and variable pH, suggests that *D*. *hansenii* could persist during extended or low‐intervention coffee fermentations. This persistence may allow sustained enzymatic activity during later fermentation stages, when aroma precursor transformation becomes increasingly important. Fourth, the bioprotective effect of volatile production observed in dairy and meat systems raises the possibility that *D*. *hansenii* could help suppress spoilage fungi or undesirable microbial overgrowth in coffee fermentation, contributing indirectly to process stability and quality consistency. This further raises the possibility that *D*. *hansenii* could suppress spoilage fungi and reduce mycotoxin accumulation in coffee fermentations, contributing to both process stability and product safety—an aspect entirely uninvestigated in coffee to date. Collectively, the current understanding of *D*. *hansenii* in coffee is entirely inferential, with no starter culture studies reported to date. It is hypothesized, based solely on extrapolated evidence, that *D*. *hansenii* could function as a targeted aroma‐modulating and bioprotective adjunct in coffee, mediating its effects through precursor hydrolysis, amino acid catabolism, phenolic biotransformation, and selective volatile synthesis rather than high ethanol productivity. Key unknowns include its competitive fitness against indigenous coffee microbiota, its glycosidase and phenolic biotransformation activity on coffee‐specific substrates, its performance across traditional and modern processing methods, and its behavior under the dynamic pH and oxygen conditions of submerged coffee fermentation. Systematic evaluation at the laboratory scale, including volatile profiling and sensory assessment across processing methods and coffee species, is urgently needed to move beyond inference and establish an evidence base for its deliberate application.

### 3.7 Further Considerations and Starter Culture Implementation

**FIGURE 5:**
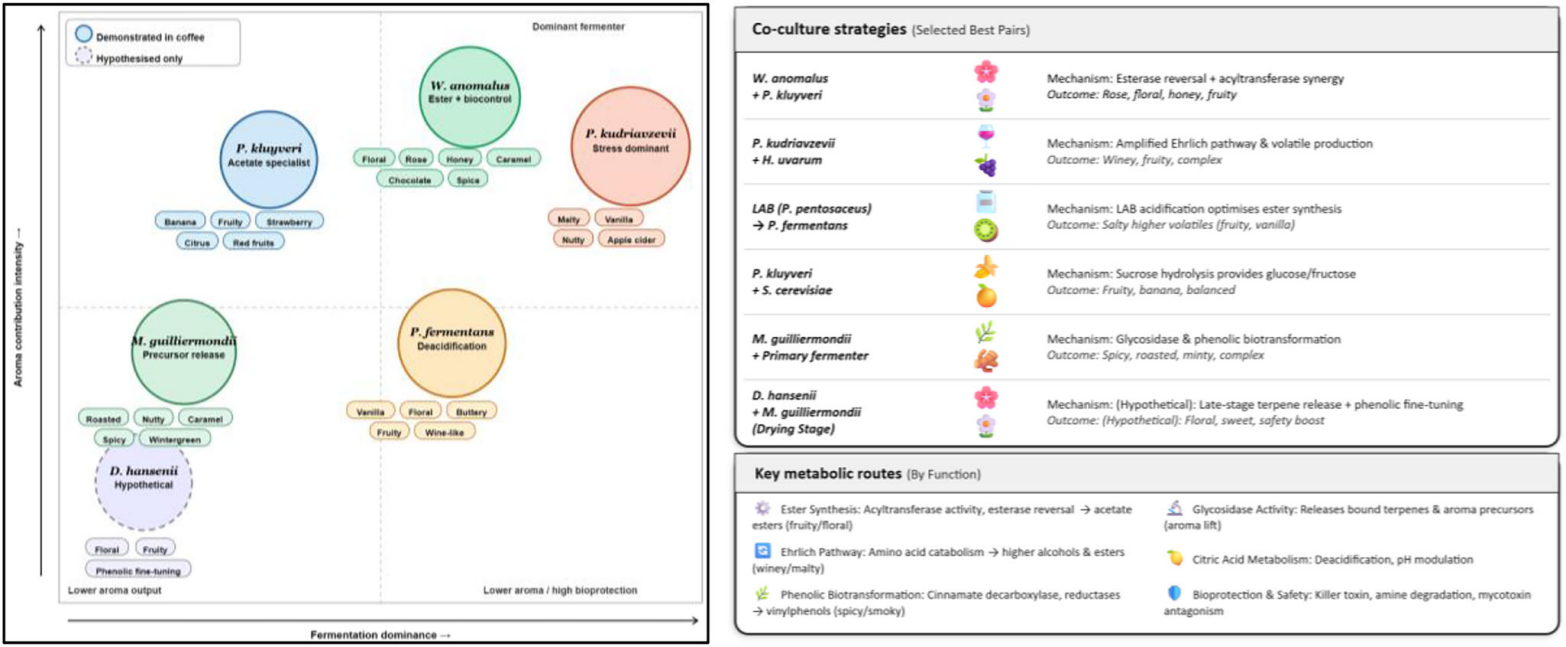
*Pichia* species flavor map for coffee fermentation. Functional positioning of six *Pichia* species mapped by fermentation dominance and aroma contribution intensity. Sensory attribute clusters indicate target cup notes associated with each species. The dashed designation for *Debaryomyces hansenii* indicates that its role in coffee fermentation is entirely hypothetical, based on extrapolated evidence from other fermented systems; no starter culture studies have been reported to date. *X*‐axis: Fermentation dominance low → adjunct/precision tool high → competitive primary fermenter; *Y*‐axis: Aroma contribution intensity low → limited/no confirmed coffee data high → strong demonstrated aroma output.

Evidence across the reviewed species consistently indicates that *Pichia* yeasts function primarily as metabolic modulators rather than dominant fermenters, with their aroma‐enhancing capacity maximized through strategic microbial pairing. Fermented coffee processing in aqueous environments involves bidirectional diffusion, whereby soluble compounds leach outward from the bean into the surrounding medium, while microbial metabolites simultaneously penetrate the bean matrix and reshape its chemical composition (Osorio et al. 2023; Shen et al. 2025; Soares et al. 2025). Co‐inoculation of *W*. *anomalus* with *P*. *kluyveri* produced the highest cupping scores across multiple trials (Mahingsapun et al. 2022), while *P*. *kudriavzevii* combined with *H*. *uvarum* enhanced volatile production beyond either species alone (Elhalis et al. 2021b). Similarly, *P*. *fermentans* paired with *P*. *pentosaceus* generated approximately threefold greater volatile content than spontaneous fermentation (de Mello Sampaio et al. 2025). Collectively, these findings reinforce a principle established in wine and beer fermentation: coculture systems should be designed according to functional complementarity rather than microbial dominance alone, pairing aroma‐modulating *Pichia* species with microorganisms that ensure efficient sugar utilization, pH control, and suppression of spoilage microbiota (Figure 5; Table S1).

The inoculation strategy represents an equally critical determinant of fermentation performance, as inoculum dose, timing, and microbial ratio exert both independent and interactive effects on metabolic outcomes. Dose‐dependent ester enhancement has been demonstrated for *P*. *kluyveri* across inoculation levels ranging from 10^5^ to 10^10^ CFU/g (Saerens and Swiegers 2014), whereas suboptimal concentrations limit metabolic contribution and excessive inoculation may promote off‐flavor accumulation. Imbalanced microbial ratios similarly reduced aromatic complexity or increased undesirable metabolites across several species (Ma et al. 2017; Taufik et al. 2024). In beverage systems, sequential inoculation—whereby *Pichia* species are introduced before *S. cerevisiae* or LAB—has consistently outperformed simultaneous inoculation by permitting early metabolic activity before competitive exclusion constrains non‐*Saccharomyces* populations. However, systematic validation of this strategy in coffee fermentation remains lacking. Importantly, protocols optimized in one fermentation matrix cannot be assumed to be transferable to another, and the absence of standardized coffee‐specific inoculation frameworks remains a major applied limitation.

The disparity between laboratory and field performance further highlights scalability as a key unresolved challenge. Mahingsapun et al. (2022) reported a decline in cupping score from 82.4 under controlled laboratory conditions to 77 at 100 kg scale, attributed to altered tank geometry, restricted aeration, and temperature variability. Maintaining inoculum viability and competitive dominance over indigenous microbiota is additionally complicated by variable cherry composition, seasonal heterogeneity, and the semi‐open nature of coffee processing relative to the closed fermentation systems used in wine and beer production. Addressing these constraints will require the development of field‐stable inoculant formulations, including lyophilized or encapsulated carriers, alongside monitoring systems capable of tracking strain persistence and metabolic activity under operational processing conditions.

Balanced assessment of *Pichia* application also requires explicit consideration of associated risks. Off‐flavor formation represents the principal concern. *W*. *anomalus* can produce ethyl acetate concentrations exceeding 150 mg/L under poorly controlled aerobic conditions, shifting its sensory contribution from desirable fruity notes to solvent‐like defects (Domizio et al. 2011). Excessive higher alcohol production has likewise been observed under imbalanced inoculation regimes across multiple species. Moreover, substantial strain‐level variation in ester production, pectinolytic activity, and stress tolerance limits the predictive value of species‐level generalizations and complicates commercial standardization. Competition with indigenous microbiota may further suppress inoculated strains or generate unpredictable metabolite profiles, as demonstrated in coffee fermentation systems (de Melo Pereira et al. 2014). Collectively, these limitations indicate that *Pichia* yeasts should be regarded as precision biotechnological tools requiring rigorous strain selection, controlled inoculation, and field‐scale validation before industrial application.

Regulatory considerations remain largely overlooked within the coffee fermentation literature despite representing a major barrier to commercial implementation. Although most *Pichia* species are considered nonpathogenic, safety assessment is fundamentally strain‐specific rather than species‐wide. *P*. *kudriavzevii* exemplifies this complexity, as certain strains have been recognized as opportunistic pathogens capable of invasive infection in immunocompromised individuals and displaying intrinsic resistance to fluconazole (Chu et al. 2023; Jamiu et al. 2021; Nguyen et al. 2024). Conversely, some *M*. *guilliermondii* strains exhibit susceptibility to clinical antifungals and lack detectable virulence determinants, supporting a nonpathogenic profile (Khanna et al. 2025). Therefore, biosafety evaluation—encompassing antimicrobial susceptibility testing, absence of virulence factors, and whole‐genome screening against curated resistance databases such as CARD—is a prerequisite for regulatory approval in most markets (Chin et al. 2024; Elhalis, See, et al. 2023; Mo et al. 2024). Although roasting is unlikely to permit the survival of inoculated yeasts, rigorous pre‐fermentation safety assessment remains necessary to minimize occupational exposure during handling and processing. The absence of a standardized regulatory framework for microbial starter cultures in coffee therefore constitutes both a practical limitation and a priority area for collaboration among researchers, industry stakeholders, and food safety authorities.

The present review is intentionally restricted to washed *C. arabica* processing, and extension to *C. canephora* (Robusta) and *Coffea liberica* represents an important research priority. Robusta generally exhibits lower aromatic complexity, higher chlorogenic acid content, and greater bitterness than Arabica (G.‐L. Hu et al. 2025; Jo et al. 2023), suggesting that ester‐producing species such as *P*. *kluyveri* and *P*. *kudriavzevii* may contribute substantially to aroma enhancement, although this hypothesis remains experimentally unverified. *C*. *liberica* presents a complementary case, often characterized by inconsistent cup quality and elevated defect incidence (Febrianto and Zhu 2026), where the bioprotective and pectinolytic activities of *Pichia* species may offer functional advantages. Furthermore, the higher phenolic load and bound aroma precursor content reported in both species (Afriliana et al. 2019; Febrianto and Zhu 2026; C. Liu et al. 2019; Murthy and Naidu 2011) suggest that glycosidase‐mediated precursor release by *M*. *guilliermondii* and ferulic acid biotransformation by *D*. *hansenii* could exert proportionally greater sensory effects than in Arabica matrices. Application in natural and honey processing systems also remains poorly explored despite their growing relevance within specialty coffee production. The distinct oxygen dynamics, prolonged cherry contact, and altered microbial succession characteristic of these processes may substantially influence *Pichia* metabolic activity and ecological competitiveness relative to washed fermentation.

An additional and insufficiently explored dimension concerns the interaction between *Pichia* starter cultures and coffee terroir—the origin‐specific sensory identity shaped by cultivar, altitude, soil composition, climate, and indigenous microbiota. A central question is whether controlled inoculation preserves, enhances, or suppresses these origin‐defining characteristics, an issue of major importance to specialty coffee markets in which geographic identity strongly influences economic value and consumer perception. Evidence from wine fermentation demonstrates that non‐*Saccharomyces* yeasts can selectively amplify or suppress varietal aroma compounds depending on strain selection and inoculation strategy (Vicente et al. 2021), suggesting that analogous dynamics may occur in coffee. Whether *Pichia* species can be selected to complement rather than override terroir expression remains unresolved. Addressing this question will require interdisciplinary research integrating metabolomics, sensory science, microbial ecology, and geographic provenance analysis across multiple cultivars and origins. Such approaches may ultimately enable the development of targeted *Pichia*‐based starter culture portfolios capable of enhancing coffee quality while preserving the origin transparency increasingly demanded within specialty coffee markets. Figure 5 and Table S1 consolidate these relationships into a structured decision‐support framework, linking species‐level metabolic activity and fermentation conditions to targeted sensory outcomes for industrial application.

## 4 Conclusion

*Pichia* yeasts constitute a functionally diverse group of non‐*Saccharomyces* microorganisms with considerable potential for controlled coffee fermentation, owing to their metabolic versatility, enzymatic capacity, stress tolerance, antifungal activity, and ability to generate aroma‐active metabolites. Critically, their contributions are neither uniform nor interchangeable. Species‐level niches are clearly differentiated: *W*. *anomalus* excels through ester synthesis and killer toxin‐based bioprotection; *P*. *kudriavzevii* dominates under physicochemical stress through Ehrlich pathway‐driven ester biosynthesis; *P*. *kluyveri* delivers disproportionate acetate ester output via species‐specific acyltransferases in coculture systems; *P*. *fermentans* contributes to deacidification and early‐stage aroma modulation; *M*. *guilliermondii* liberates bound aroma precursors through glycosidase and phenolic biotransformation; and *D*. *hansenii* contributes to ferulic acid biotransformation and biogenic amine degradation, with osmotic tolerance suggesting a distinctive role during drying stages. This specialization underscores the necessity of targeted strain selection. Contradictory outcomes across studies most often reflect differences in inoculation strategy, substrate composition, and fermentation conditions, reinforcing that knowledge transfer from wine and beer must be validated in coffee‐specific matrices. Critical gaps persist across all species: limited coffee‐specific data, absent field‐scale validation, and no systematic evaluation under anaerobic fermentation or carbonic maceration conditions. Future research should extend this framework beyond washed *Arabica* to natural and honey processing, where distinct substrate compositions and oxygen dynamics may differentially modulate *Pichia* metabolic expression. An equally important priority is elucidating how starter cultures modulate origin‐specific sensory attributes, a question central to fermentation strategies that seek to complement rather than override geographic and varietal identity in specialty coffee.

## Author Contributions

**Hosam Elhalis**: conceptualization, investigation, writing – original draft, writing – review and editing.

## Conflicts of Interest

The author declares no conflicts of interest.

## Supporting information




**Supplementary Material**: crf370607‐sup‐0001‐TableS1.docx
