# Supplementary material for: Unlock the Potential of Pichia in Coffee: Knowledge Transfer From Fermented Beverages
Source: Compr Rev Food Sci Food Saf. 2026 Aug 23;25(5):e70607. doi: 10.1111/1541-4337.70607 (PMC13500875; doi:10.1111/1541-4337.70607)
Supplement: Supplementary file 1 [file CRF3-25-e70607-s001.docx]

**Unlock the potential of *Pichia* in Coffee: Knowledge Transfer from fermented beverage**

**Hosam Elhalis^a,b*^**

^a^ Global Technology Centre, AB Mauri, Sydney, Australia; ^b^ Food Science and Technology, School of Chemical Engineering, The University of New South Wales, Sydney, Australia

*Corresponding author

Hosam Elhalis

Global Technology Centre, AB Mauri, Sydney, Australia.

Food Science and Technology, School of Chemical Engineering, The University of New South Wales, Sydney, Australia.

h.elhalis@unswalumni.com

# Table S1. *Pichia* species decision-support reference for coffee wet fermentation starter culture design.

| **Species** | **Primary functional role** | **Key metabolic pathways / mechanisms** | **Aroma-active metabolites** | **Reported or associated sensory notes** | **Recommended / reported fermentation conditions*** |
| --- | --- | --- | --- | --- | --- |
| *Wickerhamomyces anomalus* | Aroma enhancement; pectinolytic activity; bioprotection | Ester biosynthesis; extracellular pectinase and glycosidase activity; polysaccharide degradation; killer toxin and VOC-mediated biocontrol | Ethyl acetate; isoamyl acetate; 2-phenylethyl acetate | Floral, rose, honey, fruity, caramel, cocoa/chocolate, spice, citrus, apple, black tea | Wet/submerged aerobic or semi-aerobic fermentation; 10-25 °C; 10-44 h; ~10⁴-10⁷ CFU/g range reported; co-inoculation (e.g., *P. kluyveri*) enhances cupping outcomes |
| *Pichia kudriavzevii* | Stress-tolerant fermenter; aroma and volatile modulator; co-culture stabiliser | Ehrlich pathway (higher alcohols → esters); sugar metabolism under stress (low pH, heat, osmotic stress); acid modulation; hydrolytic enzyme activity inferred | Ethanol; isoamyl alcohol; phenylethyl alcohol; acetaldehyde; 3-methylbutanal; ethyl acetate; isoamyl acetate; acetoin; diacetyl | Malty, vanilla, nutty, earthy, apple cider, walnut, fruity, buttery, wine-like, chocolate, ripe cherry | Wet fermentation (aerobic/anaerobic); broad stress tolerance; ~29-31 °C reported; mixed inoculation with *H. uvarum* improves aroma; controlled ratios critical in consortia |
| *Pichia kluyveri* | Acetate ester specialist; aroma modulator; auxiliary fermenter; post-harvest biocontrol potential | Respiratory metabolism; species-specific acetyltransferase activity; amino-acid-derived ester synthesis; microbial synergy-dependent metabolism; possible competitive exclusion | Isoamyl acetate; 2-phenylethyl acetate; higher alcohols; furan-2-carbaldehyde; aldehydes | Fruity, banana, floral, citrus, red fruit, strawberry, peanut, brown sugar, caramel, chocolate, roasted, wine-like | Wet/anaerobic fermentation; 10-25 °C; 14-94 h; ~10⁵-10⁷ CFU/g (or ~5.6 log CFU/mL in studies); requires sucrose-hydrolysing partners (*S. cerevisiae*); LAB pre-fermentation enhances ester output |
| *Pichia fermentans* | Early-stage aroma modulation; deacidification; volatile producer | Glucose and citric acid metabolism; ester and aldehyde formation under moderately aerobic conditions; volatile modulation via sequential inoculation | Ethanol; ethyl acetate; isoamyl acetate; acetaldehyde; hexanal; 2,3-butanedione; phenylethyl alcohol; benzyl alcohol; benzaldehyde derivatives | Fruity, floral, vanilla, buttery, wine-like | Wet fermentation; ~28 °C; ~48 h; ~6 log CFU/mL inoculation; sequential LAB inoculation (~36 h pre-fermentation) improves volatile output; strong strain/ratio dependence |
| *Meyerozyma guilliermondii* | Bound aroma liberation; phenolic biotransformation; aroma fine-tuning (precision adjunct) | Glycosidase activity; cinnamate decarboxylation; phenolic transformation; limited pectinolytic activity; osmotic and ethanol tolerance | p-Ethylguaiacol; furfuryl alcohol; 5-methylfurfural; methyl salicylate; 2-heptanone; 2-nonanol; pyrazines | Roasted, nutty, caramel, spicy, minty, sweet; subtle aroma enhancement | Wet fermentation and SIAF; ~6.6 log CFU reported in SIAF systems; anaerobic mucilage conditions enhance volatile modulation; best as adjunct culture rather than primary inoculant |
| *Debaryomyces hansenii* | Aroma precursor hydrolysis; late-stage aroma modulation; osmotic/low-aw tolerance (adjunct role) | β-glucosidase activity; amino acid catabolism; phenolic transformation; terpene/norisoprenoid release (inferred); stress adaptation | Terpenes; norisoprenoids; higher alcohols; phenyl-derived volatiles | Floral, fruity, sweet; suppression of harsh phenolics; aroma refinement | Hypothesised role in drying/late fermentation; strong osmotic and low water activity tolerance; no coffee starter studies reported; requires experimental validation |

*Functional roles, metabolic pathways, aroma-active metabolites, target sensory attributes, and recommended fermentation conditions across the six Pichia species reviewed. D. hansenii entry is based on extrapolated evidence only; no starter culture studies in coffee have been reported to date.* Abbreviations**:** SIAF, self-induced anaerobiosis fermentation; LAB, lactic acid bacteria; CFU, colony-forming units; aw, water activity. Shading denotes individual species groupings. Dashed notation for *D. hansenii* indicates hypothetical functional role inferred from analogous fermentation systems.
